# Kinetics of light-induced mesophase transitions in azo­benzene amphiphiles containing lyotropic liquid crystals

**DOI:** 10.1107/S1600576725004923

**Published:** 2025-07-08

**Authors:** Svenja C. Hövelmann, Michael Röhrl, Ella Dieball, Michelle Dargasz, Jule Kuhn, Rajendra P. Giri, Franziska Reise, Dmytro Soloviov, Clement E. Blanchet, Michael Paulus, Thisbe K. Lindhorst, Bridget M. Murphy

**Affiliations:** a Institute of Experimental and Applied Physics Kiel University Leibnizstraße 19 24118Kiel Germany; b Ruprecht Haensel Laboratory Deutsche Elektronen-Synchrotron DESY Notkestraße 85 22607Hamburg Germany; c Otto Diels Institute of Organic Chemistry Kiel University Otto-Hahn-Platz 3-4 24118Kiel Germany; d Department Physik Universität Siegen Walter-Flex-Straße 3 57072Siegen Germany; e European Molecular Biology Laboratory, Hamburg Site c/o DESY, Notkestraße 85 22607Hamburg Germany; f Fakultät Physik/DELTA, TU Dortmund, 44221Dortmund, Germany; Montanuniversität Leoben, Austria

**Keywords:** kinetics, photoswitching, azo­benzene, lipid mesophases, vesicles, small-angle X-ray scattering, structure determination, solution scattering, structural biology

## Abstract

This study focuses on the kinetics of light-induced mesophase transitions in lyotropic liquid crystals containing a mixture of phospho­lipids and azo­benzene amphiphiles. Lipid membranes organize in a wide range of morphologies, directly influencing their functionality and the efficiency of associated components such as proteins. Transitions between mesophases occur naturally during membrane fusion and can also be triggered by multiple factors, such as pH, salinity, temperature and light. Employing light to isomerize artificial photoswitchable lipids in mixed model membranes containing 1,2-dipalmitoyl­phos­pha­tidylcholine or 1,2-didecanoyl­phosphatidylcholine revealed light-induced structural changes including mesophase transitions from a lamellar to a cubic *Pn*3*m* phase. Performing time-resolved small-angle X-ray scattering measurements, the kinetics of the change in membrane repeat distance and the transition from a lamellar to a bicontinuous cubic phase could be captured on the timescale of tens of seconds. The results demonstrate new possibilities for investigating intermediate states during mesophase transitions that are important to understand membrane fusion, and they highlight the potential of photoswitchable lipids for designing bespoke drug delivery systems.

## Abstract 2

Time-resolved small-angle X-ray scattering is applied to investigate the kinetics of reversible light-induced switching of mesophase transitions from a lamellar to a cubic *Pn*3*m* phase in mixed phospho­lipid and azo­benzene amphiphile lyotropic liquid crystals.

## 1. Introduction

Controlling the geometric shape of lyotropic lipid crystals allows one to define their structure, activity and functionality as a whole and as individual parts inside their membrane, and subsequently to influence components embedded in or interacting with the membrane structure. Natural lipid membranes are built up from a multitude of components such as phospho- and glycolipids, cholesterol, and proteins. Each cell membrane is composed of a specific combination of lipids defining its elasticity, viscosity and lipid mesophase optimized for its functional purpose in the body (Op den Kamp, 1979 ▸; Devaux, 1991 ▸; Kelley *et al.*, 2020 ▸; Lorent *et al.*, 2020 ▸). Phospho­lipids, as the main component of bilayer membranes, define the membrane mesophase of natural membranes. Upon contact with water, amphiphile lipids self-assemble and form aggregates with multiple geometric shapes and structures depending on various properties such as lipid curvature, pres­sure, pH, salinity or volume concentration. These self-assembled geometric shapes range from micelles, hexagonal structures and lamellar bilayers to twisted bilayers forming cubic mesophases (Yeagle, 2004 ▸). Bicontinuous cubic structures, with their ability to create separated water channels, are of special interest for creating ion channels (Ichikawa *et al.*, 2007 ▸), designing pharmaceutical drug delivery systems (Shah *et al.*, 2001 ▸; Hirlekar *et al.*, 2010 ▸; Martiel *et al.*, 2013 ▸) and embedding proteins to reduce their activity (Cournia *et al.*, 2015 ▸), to protect them from physical and chemical degradation (Rizwan *et al.*, 2010 ▸) or to act as an environment for protein crystallization (Cherezov *et al.*, 2002 ▸), and they are found in organelles (Walde & Ichikawa, 2021 ▸) and in cells during endo- and exocytosis (Luzzati, 1997 ▸). Each cubic structure displays distinct diffusion coefficients and modes (Zabara & Mezzenga, 2014 ▸). Therefore, controlling the lipid mesophase and its geometric shape opens new possibilities to design functional materials for pharmaceutical, food and cosmetic applications and bio-hybrid actuators and sensors (Carlsen & Sitti, 2014 ▸).

To understand the complexity of the interactions between membrane components, model membranes are employed to investigate interactions between single components. Multiple factors have been identified that can be utilized to induce a mesophase transition from a lamellar to a cubic phase and between cubic phases. Pressure (Winter *et al.*, 1998 ▸; Winter, 2002 ▸), pH (Ribeiro *et al.*, 2019 ▸) and salinity (Muir *et al.*, 2012 ▸; Kalvodova *et al.*, 2005 ▸) have been successfully employed so far. However, changing the physical or chemical properties requires specially designed sample cells, and often no reversibility between the mesophases could be reached. Therefore, we introduced in our recent study (Hövelmann *et al.*, 2024 ▸) a model system of the phospho­lipid 1,2-dipalmitoyl­phos­pha­tidyl­choline (DPPC) and a photoswitchable mimetic using light as the controlling element to induce a reversible and repeatable mesophase transition between the multilamellar and bicontinuous cubic *Pn*3*m* structures.

**Figure 1:**
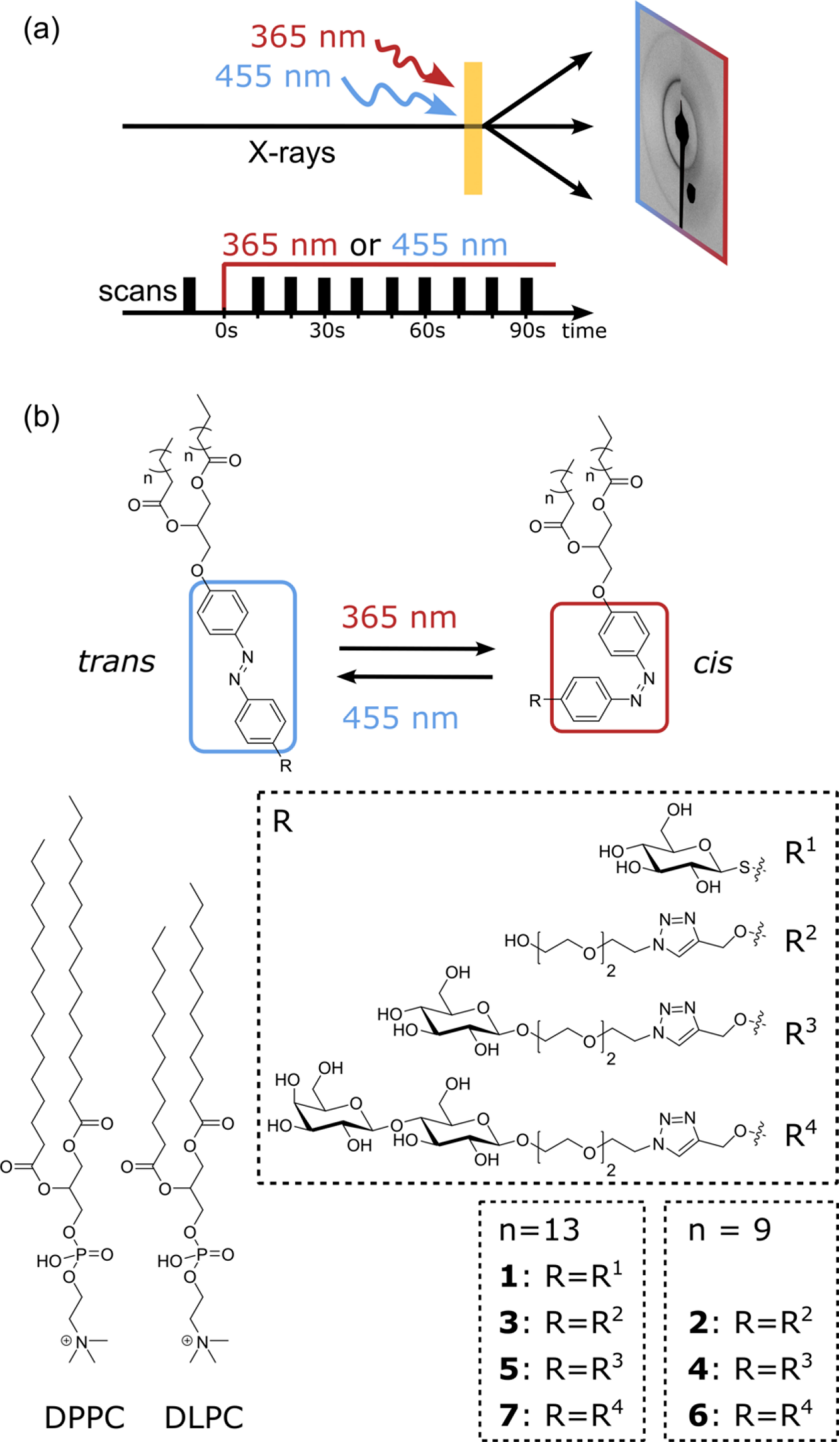
(*a*) Schematic diagram of the static (Hövelmann *et al.*, 2024 ▸) and kinetic transmission SAXS measurement setup. For the kinetic measurements, the structures were probed at various times after starting to illuminate the sample continuously as indicated. (*b*) Chemical structures of DPPC, DLPC, and both the *trans*- and *cis*-isomers of azo­benzene amphiphiles **1** to **7**. The length of the double acyl chains of the azo­benzene amphiphiles corresponds to either 12 (*n *= 9) or 16 (*n* = 13) carbon atoms.

We present an array of model systems comprising one of seven distinct photoswitchable lipid mimetics (Reise *et al.*, 2018 ▸) in combination with the phospho­lipids DPPC or 1,2-didecanoyl­phosphatidylcholine (DLPC) to identify the structures of aggregates formed by mixing azo­benzene amphiphiles and phospho­lipids in ratios varying from 0 to 100% for both components in water. In particular, the mesophase and possible light-induced mesophase transitions are of increasing interest (Fig. 1 ▸). The aggregate structures were characterized using small-angle X-ray scattering (SAXS), and time-resolved SAXS measurements were performed to investigate the evolution of the structural changes and mesophase transitions.

This research will help to design model systems for studying the influence of mesophase transitions on lipid–lipid/lipid–protein interactions and protein functionality in a light-controlled, reversible and repeatable way.

## 2. Experimental details

### 2.1. Azo­benzene mimetics

Seven azo­benzene amphiphiles, referred to as **1** to **7** hereafter as shown in Fig. 1 ▸(*b*), were investigated. Lipids **2** to **7** were synthesized in accordance with our previously published synthesis route (Reise *et al.*, 2018 ▸) and differ in the number and type of carbohydrate moieties attached (none, glucose, lactose) to the head group and in the length of the acyl chain. This being the first publication on the azo­benzene amphiphile with a glucose-based head group **1**, the synthesis route for **1** is presented in the supporting information Section S1, together with the UV–Vis absorption spectra in Fig. S10. In contrast to **2** to **7**, the glucosyl head group of **1** is linked via a sulfur atom directly to the azo­benzene photoswitch.

### 2.2. Sample preparation

DPPC and DLPC were bought from Avanti Polar lipids (Alabaster, Alabama, USA). The phospho­lipid and azo­benzene amphiphile mixtures were dissolved in 1 ml of chloro­form (Sigma Aldrich) with cumulative lipid concentrations amounting to 5 or 10 m*M* and were prepared with incrementally varying ratios of the azo­benzene amphiphiles. The mixtures were then subjected to a drying process, whereby they were reduced to thin films, using a Rotavapor R-300 from Büchi Labortechnik GmbH (Essen, Germany). The drying was conducted at a bath temperature of 45°C and a pressure of 16 mbar for a duration of at least 1 h. Subsequent to the drying process, the dried films were stored within a refrigeration unit maintained at a temperature below 10°C. On the day of the measurements, all samples were prepared by adding warm Milli-Q water to the film and then rotating and shaking the suspension in a water bath at a temperature above 45°C until a homogenous solution was formed. Larger lipid lumps were broken up using a vortex mixer and ultrasonic water bath. The pH was adjusted to 7.4 with Roti PreMix PBS salt (Carl Roth) (0.14 *M* NaCl, 2.7 m*M* KCl, 10 m*M* phosphate) for the measurement on beamline P12 at EMBL, DESY, and one of the two beamtimes on BL2 at DELTA. The hydrated samples were stored in the refrigerator at the beamline. Prior to the measurements, the hydrated solutions were allowed to reach room temperature by leaving them outside of the refrigerator for 1 h. The SAXS measurements were performed within 24 h of sample hydration. In this time frame the SAXS patterns from tested samples were identical, confirming structural stability. Samples prepared in this way are referenced as ratio lipid:number of photoswitchable mimetic, for example: 95:5 DPPC:**1**, 80:20 DPLC:**3**.

### 2.3. Isomerization

Custom-made illumination devices with rows of 365 nm LEDs [Nichia, NCSU033B(T)] and 455 nm LEDs (Osram, LD CQ7P) were employed to isomerize the azo­benzene amphiphiles from *trans* to *cis* and back. A remote connection to the illumination device was set up and the samples were illuminated for at least 5 min to switch between the *trans* and *cis* states. On beamline BL2 at DELTA, the illumination device was mounted above the capillary sample holder at a distance of about 10 cm with fluxes for 365 and 455 nm of 2.0 mW cm^−2^ in the first beamtime and 1.6 mW cm^−2^ in the second beamtime. On beamline P12 at EMBL, the illumination device was placed on top of the quartz window of the standard flow cell setup available on the beamline. A direct power measurement at the flow cell position could not be performed due to the chamber design. However, the estimated distance of 7 cm between the illumination device and the capillary would correspond to a flux of 7 mW cm^−2^. To distinguish between the *trans* and *cis* isomers, we use the naming convention *trans*-**1** and *cis*-**1**.

### 2.4. Small-angle X-ray scattering

*In situ* SAXS measurements were performed on BL2 at DELTA (Dargasz *et al.*, 2022 ▸) and on P12 at EMBL (Blanchet *et al.*, 2015 ▸) at concentrations of 10 and 5 m*M*, respectively. The corresponding mass per volume concentrations are listed in Table S1. On BL2 a simple capillary sample holder with 2 mm diameter quartz capillaries was used under an ambient atmosphere and at a room temperature of about 25°C. A photon energy of 12 keV, a beam size of about 0.6 × 0.6 mm and a MAR345 2D image-plate detector (marXperts, Norderstedt, Germany) were employed. Standard silver behenate powder was used to calibrate the detector distance and orientation. Using the *FIT2D* software (Hammersley *et al.*, 1995 ▸; Hammersley *et al.*, 1996 ▸; Hammersley, 1997 ▸; Hammersley, 2016 ▸), the 2D detector images were processed by applying a pixel mask and detector orientation correction. The data were then transformed from real to reciprocal space and an angle integration was performed to provide reduced 1D scattering patterns in *q* space [*q* = (4π/λ) sin θ, where θ is half the scattering angle and λ is the wavelength of the incident radiation]. The typical exposure time was 180 s for the data collection followed by a detector read-out time of an additional 120 s.

On the P12 EMBL BioSAXS beamline at DESY, the automated auto-sampler setup was used in combination with a PILATUS 6M detector from Dectris (Baden-Daettwil, Switzerland). Measurements were performed at 10 keV with a beam size of 0.2 × 0.12 mm. On this beamline, a flow mode for the sample is available which allows fresh sample from the stock solution to be flushed through the measurement capillary at a continuous flow rate to exchange the sample volume and reduce beam-induced damage in the sample. However, for the time-resolved measurements, the sample had to be illuminated *in situ* while being in the capillary for multiple tens of seconds. Due to the limited area of illumination, a constant sample volume was used to ensure measurement on the illuminated part. After the illumination and measurement, the sample was exchanged. After the start of the measurement, the routine was stopped during the loading process to allow manual loading of the capillary and illumination of the sample before data collection with an exposure time of 0.1 s at a transmission of 80%. Following beam damage assessment to avoid sample degradation, four frames, each with an exposure time of 0.1 s, were chosen for optimal data quality. The 2D detector images were processed automatically on the beamline and reduced 1D scattering patterns in *q* space were saved.

### 2.5. Time-resolved small-angle X-ray scattering

Time-resolved measurements were performed on the P12 EMBL BioSAXS beamline. The samples were filled into the automatic sample changer in the thermally stable *trans* state. After the sample had been loaded into the quartz capillary, there was a waiting/illumination time using the remote-controlled LEDs at either 365 or 455 nm, varying from 2 to 180 s. For the *cis* to *trans* isomerization, the sample was illuminated first at 365 nm for 60 s and then at 455 nm. During the illumination the samples were protected from X-ray damage using the fast shutter. Subsequently, the fast shutter was opened and X-ray measurements were carried out while continuing the illumination. After each scan, the capillary was rinsed automatically before being refilled for the next time delay. At each time delay, data were collected with an exposure time of 0.4 s to exclude X-ray-induced beam damage. Thus, the time resolution was set to 400 ms.

### 2.6. Analysis software

The data collected on BL2 at DELTA were analysed using a purpose-written Python script for background correction of the reduced 1D SAXS pattern and resaving the corrected pattern in the NeXus file format with associated metadata (Wilkinson *et al.*, 2016 ▸). Following the metadata standards proposed by DAPHNE4NFDI (Barty *et al.*, 2023 ▸; Lohstroh *et al.*, 2024 ▸), the newly generated NeXus file contains the background-corrected pattern, the uncorrected signal, information on the background reduction, and detector-, beamline- and sample-specific metadata. For the background reduction, reference measurements on pure water and buffer solutions were collected. The P12 data were taken as extracted by the automatic analysis pipeline of the beamline, including a background subtraction from the solvent reference measurement. Structure analysis of the BL2 and P12 data was done with custom Python scripts to determine the mesophase and *d* spacing. All SAXS data were fitted using a two-step approach, described in detail in Section S2 and by Hövelmann *et al.* (2024 ▸). The data and script are accessible from Hövelmann & Murphy (2025 ▸) and further information on their accessibility is given in the section *Data availability*.

## 3. Results and discussion

### 3.1. Tuning the mesophases

**Figure 2:**
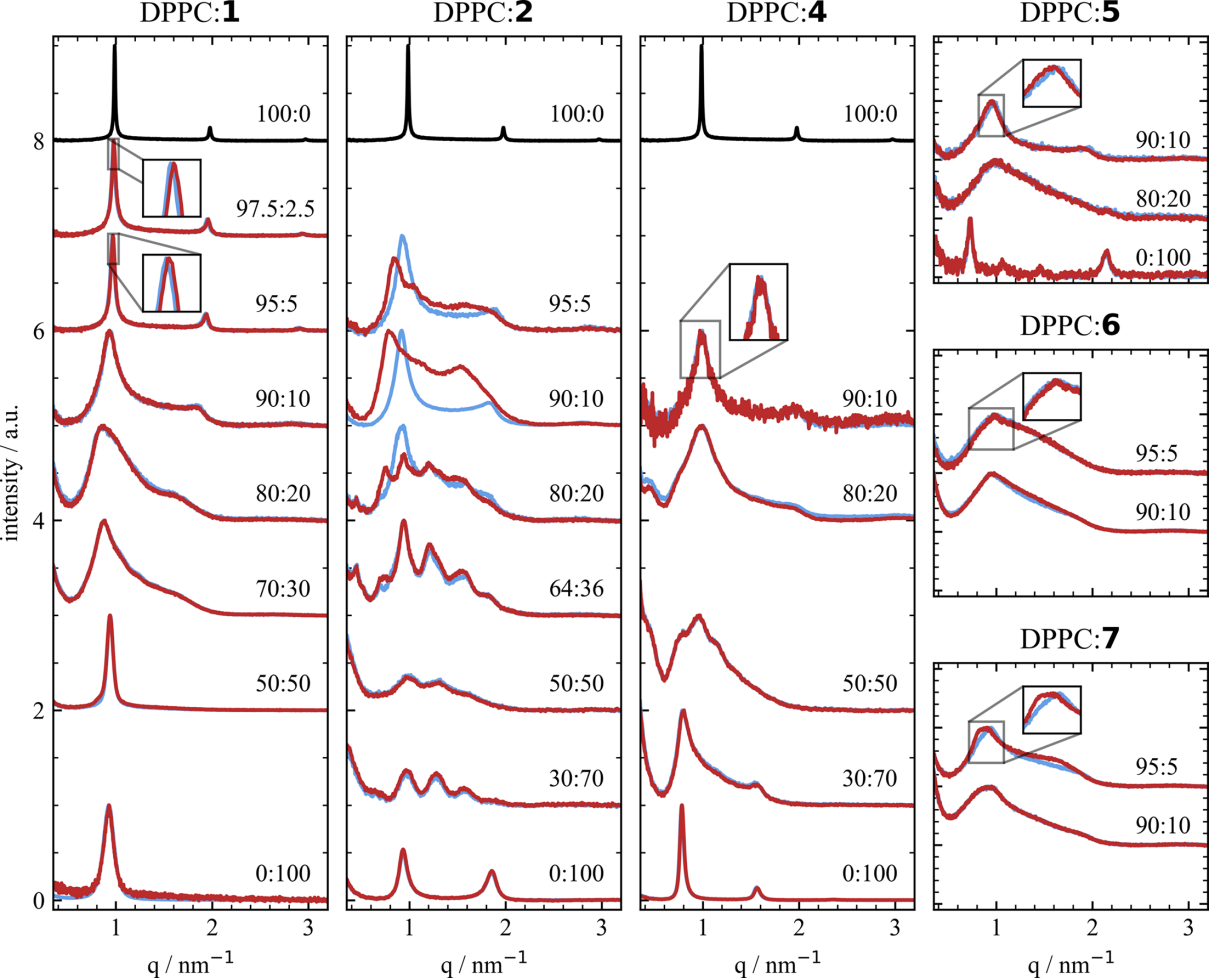
SAXS data for pure DPPC (black) and mixtures of DPPC and the azo­benzene amphiphiles **1**, **2** and **4** to **7** [Fig. 1(*b*)] in *trans* (blue) and *cis* (red) states for all mixtures in their annotated ratios. All scattering patterns shown here are background subtracted using reference measurements of the pure solution.

Investigating various combinations of DPPC or DLPC with the azo­benzene amphiphiles **1** to **7** [Fig. 1 ▸(*b*)] at 21 and 25°C with *in situ* SAXS measurements performed in the *q* range of 0.5–4.5 nm^−1^ (Fig. 2 ▸), multiple mesophases could be identified following the fitting routine described in detail in Section S2. Generally, it can be summarized that, for mixtures containing more than 20% of azo­benzene amphiphiles **2** to **7**, a bicontinuous cubic mesophase was found. For low lipid percentages, especially 2.5 and 5% of the azo­benzene amphiphiles **1** to **5**, a lamellar mesophase was observed, which corresponds to only a small deviation from the lamellar phase of pure DPPC and DLPC. For DPPC and DLPC, lamellar *d* spacings of 6.34 ± 0.03 nm and 5.89 ± 0.01 nm, respectively, were determined, which are in good agreement with previous studies (Soloviov *et al.*, 2012 ▸; Kornmueller *et al.*, 2018 ▸; Shafieenezhad *et al.*, 2023 ▸). All *d*-spacing values are listed in Table S1.

**Figure 3:**
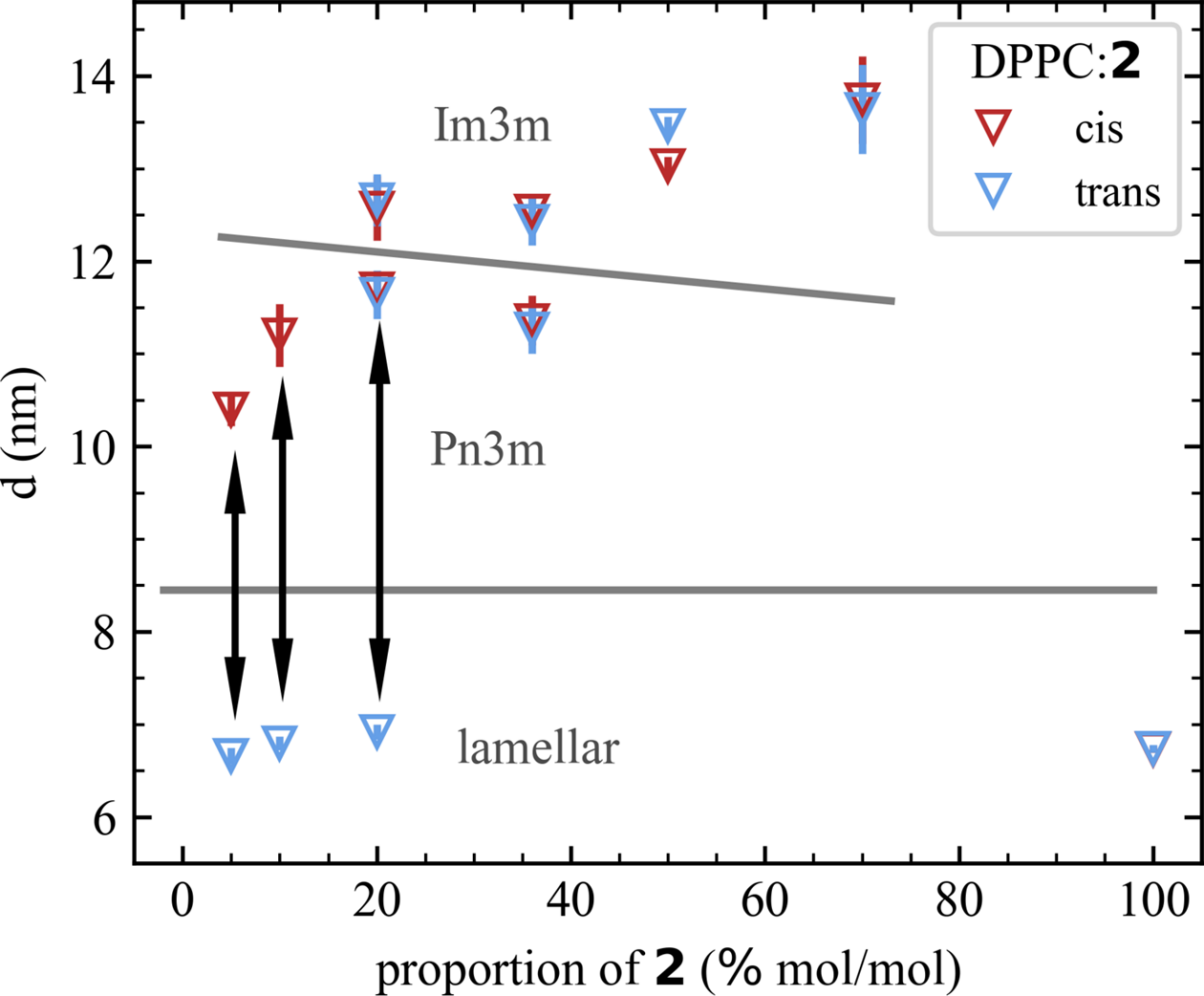
Fitted values of the *d* spacing for *trans* (blue) and *cis* (red) DPPC:**2** data. Note the increase in *d* spacing by 50% to 60% and the mesophase transition by switching from the *trans* to the *cis* state (and *vice versa*) for 95:5, 90:10 and 80:20 DPPC:**2** (highlighted by the black arrows). The grey lines are inserted as points of reference to differentiate between the different mesophases. The *d*-spacing error is estimated from the deviations of the peak positions as described in Section S2.

In mixtures of DPPC:**1** and DPPC:**2** a light-induced change in the multilayer repeat distance (*i.e. d* spacing) and switching between mesophases, respectively, were found. Upon switching from the *trans* state to the *cis* state the mesophase transition from a multilamellar phase to a bicontinuous cubic phase *Pn*3*m* was observed for DPPC:**2** (the mimetic without glycan). The kinetics of this transition are discussed below and shown in Fig. 5. The mesophase transition shows great reversibility upon switching back to the *trans* state and high reproducibility through multiple switching cycles. This finding is similar to the mesophase transition observed from a lamellar to a *Pn*3*m* phase for DPPC:**3** mixtures reported in our previous study (Hövelmann *et al.*, 2024 ▸). The azo­benzene amphiphiles **2** and **3** only differ in the length of the acyl chain [Fig. 1 ▸(*b*)]. For both DPPC:**2** and DPPC:**3**, the lamellar phase is dominant up to a content of 10% of *trans*-**2** and *trans*-**3**. On increasing the ratio of the photoswitchable lipid to 20%, cubic phases evolve in addition to the lamellar phase. For comparison, the *d* spacings and mesophases for up to 20% of **2** and **3** are listed in Table 1 ▸. While for DPPC:**3** only the additional bicontinuous cubic phase *Pn*3*m* was determined for 20% of *trans*-**3**, a coexistence of two bicontinuous cubic phases, *Pn*3*m* and *Im*3*m*, in addition to the lamellar phase was identified for DPPC:**2** for 20% of *trans*-**2**. Switching from the *trans* to the *cis* state results in the disappearance of the lamellar phase peaks and an increase in intensity of the peaks belonging to the *Pn*3*m* structure, while the intensity and position of the *Im*3*m* peaks remain constant. This suggests that the mesophase transition only takes place between the lamellar and *Pn*3*m* structured parts, while the sections structured in *Im*3*m* are unaffected by the conformational change of **2**. Identification of the phases has been performed by checking combinations of different phases to match the peaks visible in the SAXS data (Fig. S11). At percentages higher than 36% of **2**, no difference between the *trans* and *cis* states was observed and the mesophase was identified to be a coexistence of *Pn*3*m* and *Im*3*m* phases. At 70% of **2**, while both the *Pn*3*m* and *Im*3*m* phases give a reasonable fit (Fig. S12), the fitted values for the *Im*3*m* phase are more reasonable as the *d* spacing is close to that for the *Im*3*m* structure observed at 50% and below (Table S1). Therefore, in Fig. 3 ▸ only the fitted value for the *Im*3*m* structure is included. Increasing the percentage of *trans*-**2** to 100% reveals the formation of multilamellar vesicles with a spacing of 6.74 ± 0.05 nm. The *d*-spacing values are shown graphically in Fig. 3 ▸ to visualize the switching-induced mesophase transition and change in *d* spacing.

Whereas DPPC:**2** and DPPC:**3** both show light-induced mesophase transitions, combinations of DPPC with the sugar-containing azo­benzene amphiphiles **1**, **5** and **7** display an increase or decrease in the *d* spacing upon switching. In contrast, for combinations of DPPC with the shorter chain sugar-containing mimetics **4** and **6** no light-induced structural change could be observed. Both 90:10 DPPC:**5** and 95:5 DPPC:**7** mixtures show a shift of the first- and second-order peaks to smaller *q* upon switching to the *cis* state, corresponding to an increase in the *d* spacing and an expansion of the structure from 6.42 ± 0.08 nm to 6.57 ± 0.08 nm for the 90:10 DPPC:**5** sample. For 100% of **5**, two lamellar lipid phases with *d* spacings of 8.63 ± 0.01 nm and 5.89 ± 0.02 nm are found. Unfortunately, the exact geometric structure for 80:20 DPPC:**5** could not be determined, as the lack of clear peaks leads to reasonable fits for multiple cubic structures and any combination thereof. The same issue occurs for the data of DPPC:**6** and DPPC:**7**. Nevertheless, all identified structures and *d* spacings are listed in Table S1. In contrast to the increase in *d* spacing for 90:10 DPPC:**5** and 95:5 DPPC:**7**, the data for both 97.5:2.5 and 95:5 DPPC:**1** show a decrease in the lattice parameter by 0.04 nm upon switching to the *cis* state. Having both a glucose-based head group and the same acyl chain length, **1** and **5** differ in the linker between the azo­benzene and glucose groups [Fig. 1 ▸(*b*)]. Above 10% no difference between the *trans*-**1** and *cis*-**1** states is observed. At higher percentages than 20% of **1**, another structure adds to the lamellar phase structure, and from 50% upwards only a first-order peak with a repeat distance of 6.67 ± 0.01 nm and no higher-order peaks are detected.

Overall, the data illustrate the strong dependency of the mesophase on the structure of the azo­benzene amphiphile and show that mixtures with shorter azo­benzene amphiphiles form more defined mesophases. Especially for the longest, lactose-containing, azo­benzene amphiphiles **6** and **7**, the solubility during sample preparation decreases greatly at higher percentages and the scattering signal is less defined, with the absence of clear peaks complicating the mesophase determination. Further, we found that a small but visible change in structure upon switching was observed for **5** and **7**, although the comparable molecules **4** and **6**, with a shorter acyl chain but identical head group, showed no structural difference. Also, the percentage up to which a switching could be observed decreases with the length of the photoswitchable molecule. While photoswitching was detected for percentages of up to 20% of **2** and **3** (both with no sugar), for **5** (glucose) and **7** (lactose) a difference between the *trans* and *cis* states was only found up to 10% and 5%, respectively.

To identify the effect of the phospho­lipid present in the liquid crystal on the light-induced mesophase transition, we investigated mixtures of DLPC with **2** and **3**. Compared with DPPC, DLPC possesses shorter acyl chains [Fig. 1 ▸(*b*)], and it is in the liquid crystalline phase at room temperature as its phase transition temperature of −2°C (Mabrey & Sturtevant, 1976 ▸) is much lower than that for DPPC (41°C; Biltonen & Lichtenberg, 1993 ▸).

**Figure 4:**
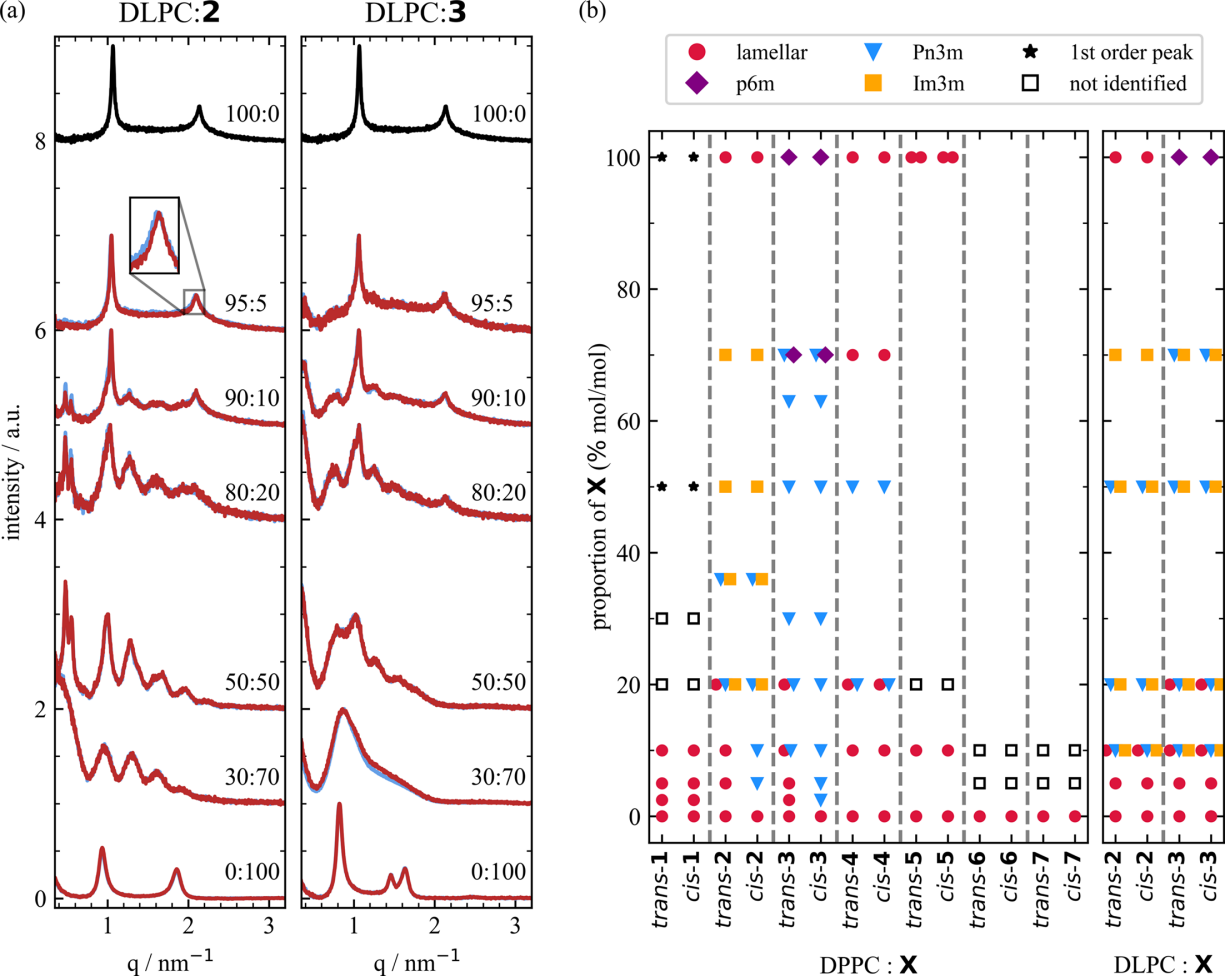
(*a*) SAXS data for pure DLPC (black) and mixtures of DLPC and the azo­benzene amphiphiles **2** and **3** in *trans* (blue) and *cis* (red) states. (*b*) Mesophases for mixtures of DPPC, DLPC and the azo­benzene amphiphiles **1** to **7** [Fig. 1(*b*)]. Mesophases for DPPC:**3** are taken from Hövelmann *et al.* (2024 ▸).

Similar to the mixtures with DPPC, the DLPC mixtures show a lamellar mesophase for low percentages of **2** and **3** as shown in Fig. 4 ▸(*a*). The lamellar *d* spacings for 95:5 DLPC:**2** and 95:5 DLPC:**3** are 6.00 ± 0.01 nm and 5.93 ± 0.01 nm, respectively, smaller than their DPPC-containing counterparts. Yet the absolute increase in the *d* spacing upon adding 5% of **2** and **3** is comparable for DLPC and DPPC. At 10%, coexisting cubic phases *Pn*3*m* and *Im*3*m* emerge and above 20% only cubic phases are present. Despite the similarities, only a very small increase in the lateral structural parameter within the error bars is observed for 5% of **2**, while for all higher percentages of **2** and all percentages measured of **3** no structure difference upon switching is found. Most likely, the lack of a structural change upon illumination stems from the difference in phase of the phospho­lipids. Being in the liquid crystalline phase, the DLPC molecules are more flexible and their fluidity is higher than the DPPC molecules, suggesting that photoswitching does not result in an observable change of the mesophase. In previously conducted measurements at temperatures above the phase transition of DPPC, no structural change was observed for DPPC:**3** mixtures either (Hövelmann *et al.*, 2024 ▸), strongly endorsing the dependence of the observed mesophase change on the lipid phase. The diversity of mesophases found for all the mixtures of liquid crystals reported here is summarized graphically in Fig. 4 ▸(*b*).

### 3.2. Kinetics of switching

**Figure 5:**
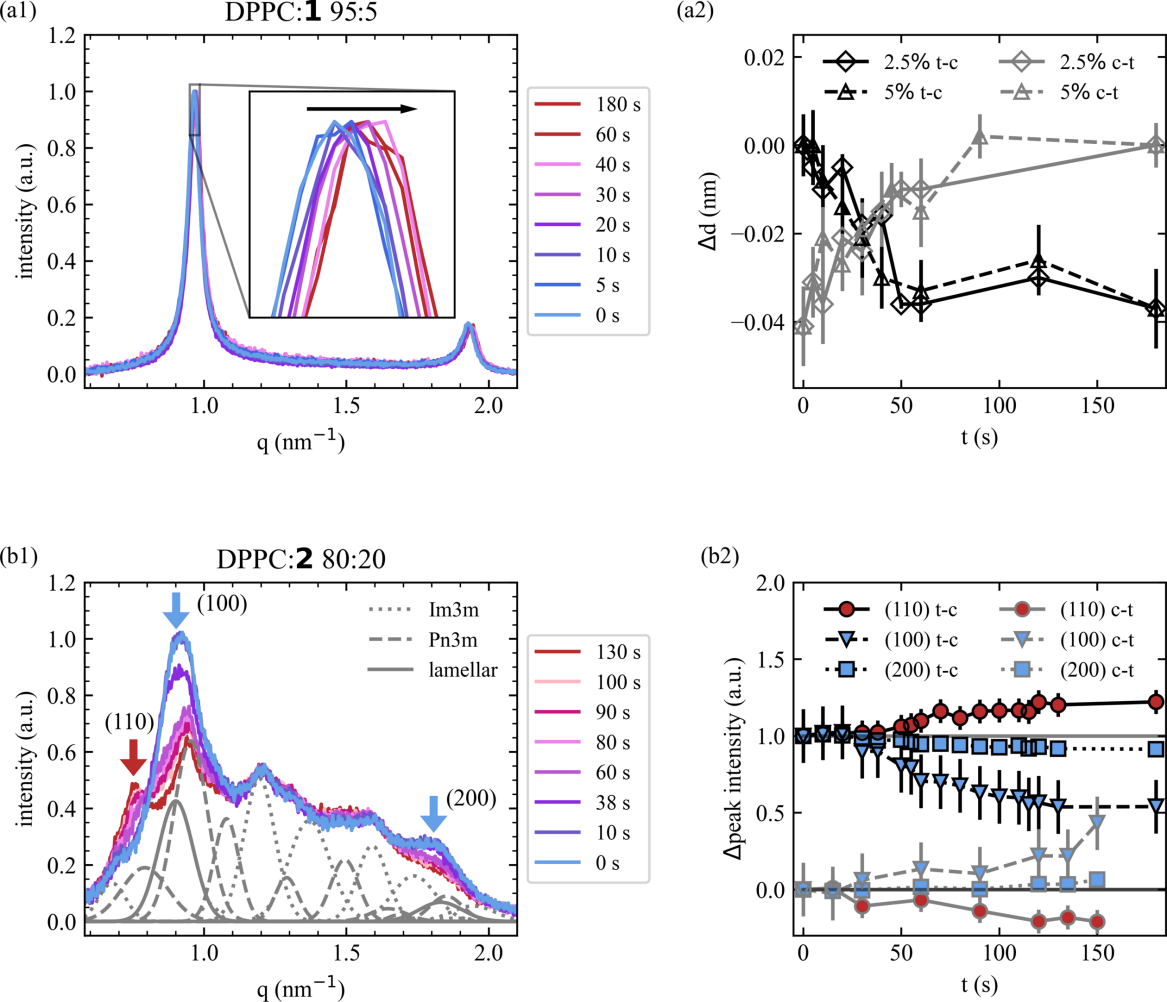
Time-resolved SAXS data for azo­benzene amphiphile mixtures of (*a*1) 95:5 DPPC:**1** and (*b*1) 80:20 DPPC:**2** from the *trans* state (blue) to the *cis* state (red) after illuminating the sample for the given number of seconds. In addition to the measured scattering data, the Gaussian curves belonging to the fitted lamellar (blue), *Pn*3*m* (red/grey solid lines) and *Im*3*m* (grey dotted lines) phases for the *trans* state at 0 s are shown underneath the data in panel (*b*1) (the fits for the *cis* state are shown in Fig. S11). (*a*2) Fitted values of the *d* spacing for 95:5 and 97.5:2.5 DPPC:**1**. (*b*2) Comparison of the intensity of the lamellar phase 100 and 200 peaks and *Pn*3*m* 110 peak [marked by arrows in panel (*b*1)] of 80:20 DPPC:**2** for the *trans* to *cis* (t-c) and *cis* to *trans* (c-t) isomerization.

Time-resolved measurements were performed on 97.5:2.5 and 95:5 DPPC:**1** and 80:20 DPPC:**2** samples on the EMBL beamline P12. Utilizing the automatic sample changer on the beamline, the sample was pre-illuminated at 365 and 455 nm to bring the sample to the *trans* or *cis* state, respectively. To achieve a time delay between illumination and X-ray irradiation, the sample was loaded manually into the sample capillary, as described in the experimental details of the time-resolved SAXS measurements, Section 2.5. For each sample, two runs with multiple measurements were performed to capture the light-induced structure change *in situ* for both transitions from *trans* to *cis* and *vice versa*. A selection of scans for 95:5 DPPC:**1** and 80:20 DPPC:**2** are shown in Figs. 5 ▸(*a*1) and Fig. 5 ▸(*b*1), respectively. All scans were fitted with the same parameters as for the structures found from the static measurements. For DPPC:**1**, a decrease and increase in the *d* spacing can be observed upon switching from *trans* to *cis* and *vice versa*. While the lamellar mesophase stays constant, the *d* spacing increases and decreases by about 0.03 nm for 2.5:97.5 and 5:95 DPPC:**1**. The thickness starts to change within the first 5 s of illumination at 455 and 365 nm, and reaches the final thickness for the *trans* and *cis* states after 50 s for 2.5% of **1**. Similarly, for 5% of **1** the *cis* state is reached after 40 s, while switching back to the *trans* state takes about 90 s. The fitted *d* spacings are shown in Fig. 5 ▸(*a*2).

In contrast to the instantaneous induced thickness increase and decrease observed for both DPPC:**1** samples, the light-induced mesophase transition found in 80:20 DPPC:**2** does not occur immediately. The first observable change in structure takes place after 30 s of illuminating the sample. For the *trans* state, peaks belonging to three mesophases were identified, namely the lamellar, cubic *Pn*3*m* and *Im*3*m* phases as listed in Table 1 ▸. The fits revealed that the *d* spacings for all three phases stayed constant during isomerization within the error bars (Table S2). Comparing the peaks of the *trans* and *cis* states, the peaks belonging to the *Im*3*m* structure do not change in either position or relative intensity. This suggests that the *Im*3*m* structure is unaffected by the structural re­arrangement and the switching only happens between the lamellar and cubic *Pn*3*m* structures. Therefore, each scan was normalized to the *Im*3*m* 211 peak intensity. In the *trans* state the first- (100) and second-order (200) peaks belonging to the lamellar phase are prominent but they disappear when switching to the *cis* state. Meanwhile, the 110 peak of the *Pn*3*m* structure evolves strongly upon switching to the *cis* state [Fig. 5 ▸(*b*1)]. To follow the temporal development of the phase transition, the intensities of the 100, 200 and 110 peaks are visualized in Fig. 5 ▸(*b*2). Isomerizing from the *trans* to the *cis* state, the first changes in intensity of the 100 and 200 peaks are observed after 30 s of illumination at 365 nm. Meanwhile, the intensity of the 110 peak stays constant and starts to increase at 50 s. Though the start of the appearance of the 110 peak is delayed compared with the disappearance of the 100 and 200 peaks, all peaks reach their final intensity after 120 s. This suggests that a certain number of molecules have to be isomerized before a phase transition is induced. Switching back from the *cis* to the *trans* state, the change in peak intensity is observed after 30 s of illumination at 455 nm for all three peaks simultaneously. This suggests the mesophase transition from *Pn*3*m* to a lamellar phase upon isomerization back to the *trans* state is faster and may have a different mechanism. Nevertheless, the mesophase transition takes 120 s to reach equilibrium.

## 4. Conclusion

In this work, we confirmed with small-angle X-ray scattering that visible and UV light is able to induce reversible and repeatable structural changes in lyotropic liquid crystals. The crystals consist of mixtures of DPPC, DLPC and photoswitchable azo­benzene amphiphiles, named **1** to **7**, in aqueous solutions. Generally, photoswitching of the mesophase was observed for percentages up to 20% of azo­benzene molecules **2** without a carbohydrate group, **1** and **5** both with glucose, and **7** with lactose attached to the headgroup in combination with DPPC at room temperature. Similarly to our previous investigation on the combination of DPPC and mimetic **3** without a carbohydrate head group (Hövelmann *et al.*, 2024 ▸), a light-induced mesophase transition from a lamellar structure in the *trans* state to a cubic *Pn*3*m* structure in the *cis* state was observed for DPPC:**2** mixtures for a percentage of up to 20% of **2**. For sugar-containing mimetics **1**, **5** and **7**, small changes in the lamellar *d* spacing were detected up to lipid percentages of 5%, 10% and 5%, respectively. All structural changes and mesophase transitions could be switched reversibly, repeatedly and reproducibly. While structural changes were observed for multiple combinations with DPPC, lyotropic liquid crystals consisting of DLPC and photoswitchable molecules showed no light-induced changes, suggesting that the overall membrane properties influenced by the phospho­lipids play a vital role in whether a structural change is observed upon isomerization of the azo­benzene amphiphile.

Performing time-resolved SAXS measurements allowed the kinetics of the light-induced structural changes to be observed *in situ* for DPPC:**1** and DPPC:**2** mixtures. For the glucose-containing DPPC:**1**, the lamellar *d* spacing starts to change within seconds of the initial illumination and reaches its final value within 90 s. Meanwhile, the light-induced mesophase transition from a lamellar to a *Pn*3*m* phase in 80:20 DPPC:**2** (no sugar) happened on the minute timescale. The transition was tracked by comparing the intensities of the 100 and 200 peaks belonging to the lamellar phase and the cubic *Pn*3*m* 110 peak. While the intensity of the lamellar peaks starts to decrease after 30 s of illumination for isomerization into the *cis* state, the *Pn*3*m* peak only rises after 50 s of illumination. Meanwhile, during *trans* isomerization, the *Pn*3*m* peak and lamellar peaks change their intensity simultaneously after 30 s. After 120 s the isomerization is complete for both the isomerization from *cis* to *trans* and back.

These findings indicate that the combination of DPPC and azo­benzene amphiphiles in lyotropic liquid crystals is optimal to observe light-induced mesophase transitions at room temperature. The type of induced structural changes depends on the specific azo­benzene amphiphile. The timescale for light-induced mesophase transitions has been identified as being in the multiple tens of seconds. Investigating these phase transitions and the timescale of their kinetics furthers the understanding of cellular fusion processes and allows for the design and evaluation of membrane systems for drug delivery and protein folding.

## 5. Related literature

For further literature related to the supporting information, see Bruneau *et al.* (2015 ▸), Dams *et al.* (2013 ▸) and Leriche *et al.* (2010 ▸).

## Supplementary Material

Synthesis details. DOI: 10.1107/S1600576725004923/xx5073sup1.pdf

## Figures and Tables

**Table:** 

*T* (°C)	DPPC:**1**	Mesophase	*d* (nm)
25	100:0	Lamellar	6.35 ± 0.01

**Table:** 

		*trans*		*cis*	
*T* (°C)	DPPC:**1**	Mesophase	*d* (nm)	Mesophase	*d* (nm)
21	97.5:2.5	Lamellar	6.44 ± 0.01	Lamellar	6.40 ± 0.02
21	95:5	Lamellar	6.51 ± 0.01	Lamellar	6.47 ± 0.02
21	90:10	Lamellar	6.71 ± 0.04	Lamellar	6.75 ± 0.04

**Table:** 

		*trans*		*cis*	
*T* (°C)	DPPC:**2**	Mesophase	*d* (nm)	Mesophase	*d* (nm)
25	95:5	Lamellar	6.7 ± 0.09	*Pn*3*m*	10.4 ± 0.2
25	90:10	Lamellar	6.8 ± 0.08	*Pn*3*m*	11.2 ± 0.4
21	80:20	*Im*3*m*	12.7 ± 0.3	*Im*3*m*	12.6 ± 0.4
		*Pn*3*m*	11.6 ± 0.3	*Pn*3*m*	11.7 ± 0.2
		Lamellar	6.9 ± 0.09		

**Table:** 

		*trans*		*cis*	
*T* (°C)	DPPC:**3**	Mesophase	*d* (nm)	Mesophase	*d* (nm)
25	97.5:2.5	Lamellar	6.42 ± 0.03†	*Pn*3*m*	11.1 ± 0.6†
25	95:5	Lamellar	6.45 ± 0.03†	*Pn*3*m*	11.1 ± 0.4†
25	90:10	Lamellar	6.55 ± 0.03†	*Pn*3*m*	11.2 ± 1.0†
		*Pn*3*m*	11.0 ± 0.7†		
25	80:20	Lamellar	6.53 ± 0.05†	*Pn*3*m*	10.4 ± 1.0†
		*Pn*3*m*	11.4 ± 0.7†		

† Reference values for DPPC:**3** from Hövelmann *et al.* (2024 ▸).

## Data Availability

Raw data, fit parameters and analysis scripts are available via https://doi.org/10.57892/100-112. International generic sample numbers (IGSNs): DPPC (https://doi.org/10.60578/x2ep-qgqj); DPPC:**1** 97.5:2.5 (https://doi.org/10.60578/pmzq-9at9); DPPC:**1**95:5 (https://doi.org/10.60578/s4fn-nmc1); DPPC:**1** 90:10 (https://doi.org/10.60578/k080-8a1z); DPPC:**1** 80:20 (https://doi.org/10.60578/32vk-hk7k); DPPC:**1** 70:30 (https://doi.org/10.60578/na1d-eqhc); DPPC:**1** 50:50 (https://doi.org/10.60578/yf0e-mqt6); DPPC:**1** 0:100 (https://doi.org/10.60578/27sy-9a6j); DPPC:**2** 95:5 (https://doi.org/10.60578/u26c-tzwe); DPPC:**2** 90:10 (https://doi.org/10.60578/hfys-47sn); DPPC:**2** 80:20 (https://doi.org/10.60578/hr19-er73); DPPC:**2** 64:36 (https://doi.org/10.60578/wqv9-2pu7); DPPC:**2** 50:50 (https://doi.org/10.60578/3hj1-g8a4); DPPC:**2** 30:70 (https://doi.org/10.60578/a4s8-76vt); DPPC:**2** 0:100 (https://doi.org/10.60578/gs8q-11sm); DPPC:**4**90:10 (https://doi.org/10.60578/f3dp-378u); DPPC:**4** 80:20 (https://doi.org/10.60578/64b0-d2ch); DPPC:**4** 50:50 (https://doi.org/10.60578/fzju-5yay); DPPC:**4** 30:70 (https://doi.org/10.60578/1fe1-mp5k); DPPC:**4** 0:100 (https://doi.org/10.60578/mghe-0vqg); DPPC:**5** 90:10 (https://doi.org/10.60578/51s4-vskp); DPPC:**5** 80:20 (https://doi.org/10.60578/r9qu-m53n); DPPC:**5**0:100 (https://doi.org/10.60578/2pcd-7u3b); DPPC:**6** 95:5 (https://doi.org/10.60578/nfs5-qxw2); DPPC:**6** 90:10 (https://doi.org/10.60578/gs1e-7wrk); DPPC:**7** 95:5 (https://doi.org/10.60578/ujbp-4acv); DPPC:**7** 90:10 (https://doi.org/10.60578/6d37-2h53); DLPC (https://doi.org/10.60578/x9e6-y42z); DLPC:**2** 95:5 (https://doi.org/10.60578/pv02-4bj7); DLPC:**2**90:10 (https://doi.org/10.60578/1ekt-pwaa); DLPC:**2** 80:20 (https://doi.org/10.60578/w37y-tubw); DLPC:**2** 50:50 (https://doi.org/10.60578/9xk4-0a1c); DLPC:**2** 30:70 (https://doi.org/10.60578/8ebr-p7hs); DLPC:**3** 95:5 (https://doi.org/10.60578/87qy-qbhc); DLPC:**3** 90:10 (https://doi.org/10.60578/pkfk-rqw6); DLPC:**3** 80:20 (https://doi.org/10.60578/2vxt-bbqh); DLPC:**3**50:50 (https://doi.org/10.60578/vwun-1p59); DLPC:**3** 30:70 (https://doi.org/10.60578/syvh-v1wf).
