# Supplementary material for: Kinetics of light-induced mesophase transitions in azo­benzene amphiphiles containing lyotropic liquid crystals
Source: J Appl Crystallogr. 2025 Jul 8;58(Pt 4):1322–31. doi: 10.1107/S1600576725004923 (PMC12321011; doi:10.1107/S1600576725004923)
Supplement: Supplementary file 1 [file j-58-01322-sup1.pdf]

JOURNAL OF APPLIED CRYSTALLOGRAPHY

Volume 58 (2025)

Supporting information for article:

Kinetics of light-induced mesophase transitions in azobenzene amphiphiles containing lyotropic liquid crystals

Svenja C. Hövelmann, Michael Röhrl, Ella Dieball, Michelle Dargasz, Jule Kuhn, Rajendra P. Giri, Franziska Reise, Dmytro Soloviov, Clement E. Blanchet, Michael Paulus, Thisbe K. Lindhorst and Bridget M. Murphy

S1. Synthesis route of 1

S1.1. General Information

S1.1.1. Chemicals

The reagents employed in the described syntheses are commercially available unless otherwise noted. (4-Hydroxyphenyl)(4'-iodophenyl)diazene (**8**) (Leriche *et al.*, 2010), solketal tosylate (**9**) (Dams *et al.*, 2013) and 1-thio-β-D-glucopyranose (**10**) (Bruneau *et al.*, 2015) were synthesized according to the literature procedures annotated. Dry solvents were either purchased directly (1,4-dioxane, DMF), or in the case of chloroform dried over molecular sieves 4 Å according to standard procedures. For extractions and column chromatography, technical grade solvents were used, which were purified by distillation before use.

S1.1.2. NMR spectroscopy

^{1}H and ^{13}C NMR spectra were recorded on a Bruker Avance (600 MHz) NMR spectrometer at 298 K. The ^{1}H NMR shifts were determined using the solvent peaks as reference: $CDCl_3$ (^{1}H = 7.26 ppm, ^{13}C 77.2 ppm) or DMSO-d_6 (^{1}H = 2.50, ^{13}C = 39.5 ppm). Data are presented in the following format: chemical shift, multiplicity (s = singlet, d = doublet and m = multiplet), coupling constant J in Hertz (Hz) and integration. Structural assignment of signals was performed utilizing 2D-NMR spectroscopy (COSY, HSQC, HMBC).

S1.1.3. Infrared spectroscopy

The infrared (IR) spectra were measured with a PerkinElmer FT-IR Spectrum Two (UATR) spectrometer and are reported in cm^{-1}.

S1.1.4. Thin layer chromatography (TLC)

TLC was performed on silica gel plates (GF 254, Merck). Visualization was achieved by either a vanillin-(3.0 g vanillin and 0.5 mL H_2SO_4 in 100 mL EtOH) or $KMnO_4$-based staining solution (1.5 g $KMnO_4$, 10 g K_2CO_3 and 1.25 mL 10% NaOH solution (aq.) in 200 mL water) followed by heat treatment at approximately 200 °C.

S1.1.5. Flash chromatography

The products were purified by flash chromatography on silica gel columns (Merck, 230–400 mesh, particle size 0.040–0.063 mm) or by automated flash chromatography using a puriFlash450 or puriFlash5.020 device (Interchim®).

S1.1.6. MALDI/ESI MS spectrometry

HR-ESI mass spectra were recorded on a ThermoFischer Orbitrap mass spectrometer. Samples were prepared using a solvent mixture of acetonitrile/ water (4:1) containing ammonium formate as an additive in a concentration of 1.3 mM.

S1.2. Synthesis

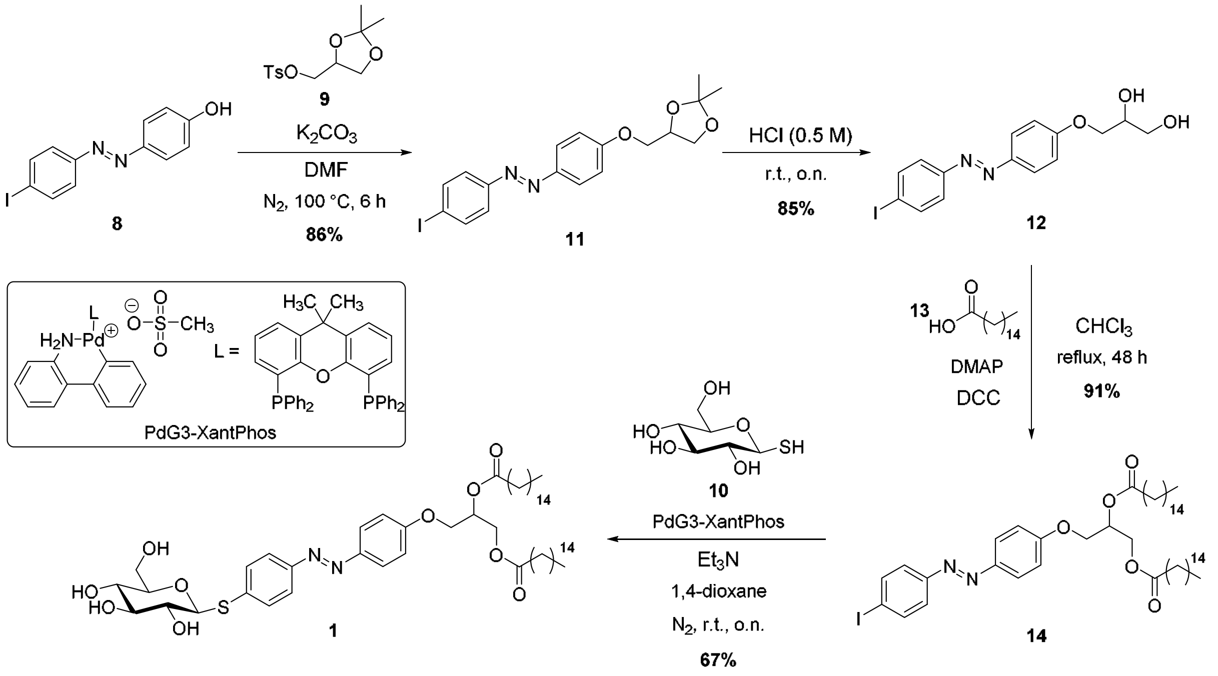

Figure S1 Synthetic overview for the preparation of the photoswitchable glycolipid mimetic **1**. Starting from (4-hydroxyphenyl)(4'-iodophenyl)diazene (**8**) the glycerol moiety is introduced in the form of the tosyl-activated solketal **9**, followed by deprotection of the isopropylidene protecting group to result in the azobenzene glycerol derivate **12**. The lipid tail of the mimetic is completed by esterification with hexadecanoic acid (**13**) resulting in the diacyl glycerol **14**. In the final step, a modified Buchwald-Hartwig-Migita coupling derived from a method published by (Bruneau *et al.*, 2015) was performed to introduce the hydrophilic head group 1-thio-β-D-glucopyranose (**10**) resulting in target glycolipid mimetic **1**. DCC: *N*,*N*'-dicyclohexylcarbodiimide; DMAP: *N*,*N*-dimethylpyridin-4-amine; DMF: *N*,*N*-dimethylformamide.

1-*O*-(4-(4-Iodophenyldiazenyl)benzene)-2,3-di-*O*-isopropylidene-*rac*-glycerol (**11**):

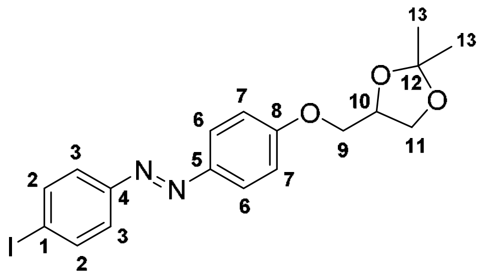

(4-Hydroxyphenyl)(4'-iodophenyl)diazene (**8**) (2.00 g, 6.17 mmol, 1.00 equiv) was dissolved in dry DMF under N_2-atmosphere. Solketal tosylate (**9**) (1.80 g, 6.29 mmol, 1.02 equiv) and potassium carbonate (2.03 g, 14.7 mmol, 2.38 equiv) were added. The reaction mixture was heated to 100 °C for 6 h under stirring and monitored by TLC. The solvent was removed under reduced pressure. The residue was taken up in ethyl acetate (300 mL) and the organic phase washed with water (100 mL) and brine (100 mL). The organic phase was dried over $MgSO_4$, it was filtered and the solvent removed under reduced pressure. The product was purified by column chromatography (cyclohexane/ ethyl acetate 8:1). 1-*O*-(4-(4-Iodophenyldiazenyl)benzene)-2,3-di-*O*-isopropylidene-*rac*-glycerol (**11**) (2.32 g, 5.29 mmol, 86% yield) was isolated as an orange solid.

R_f = 0.53 (cyclohexane/ ethyl acetate 4:1).

IR (ATR): $\tilde{\nu}$ = 2973, 2893, 1602, 1584, 1041, 838, 543.

^{1}H NMR (600 MHz, $CDCl_3$) δ 7.92 – 7.88 (m, 2H, H-6), 7.86 – 7.82 (m, 2H, H-2), 7.63 – 7.59 (m, 2H, H-3), 7.05 – 7.01 (m, 2H, H-7), 4.52 (dddd, $^3J_{\text{H-10–H-11a}}$ = 6.5 Hz, ^3J = 5.7, 5.7, 5.7 Hz, 1H, H-10), 4.19 (dd, $^2J_{\text{H-11a–H-11b}}$ = 8.5 Hz, $^3J_{\text{H-11a–H-10}}$ = 6.4 Hz, 1H, H-11a), 4.14 (dd, $^2J_{\text{H-9a–H-9b}}$ = 9.5 Hz, $^3J_{\text{H-9a–H-10}}$ = 5.4 Hz, 1H, H-9a), 4.04 (dd, $^2J_{\text{H-9b–H-9a}}$ = 9.5 Hz, $^3J_{\text{H-9b–H-10}}$ = 5.8 Hz, 1H, H-9b), 3.93 (dd, $^2J_{\text{H-11b–H-11a}}$ = 8.5 Hz $^3J_{\text{H-11b–H-10}}$ = 5.8 Hz, 1H, CH_2b'), 1.48, 1.42 (each s, each 3H, 3 CH_3-13).

^{13}C NMR (151 MHz, $CDCl_3$) δ 161.4 (1C, C-8), 152.2 (1C, C-4), 147.2 (1C, C-5), 138.4 (2C, C-2), 125.1 (2C, C-6), 124.4 (2C, C-3), 115.0 (2C, C-7), 110.1 (1C, C-12), 97.0 (1C, C-1), 74.0 (1C, C-10), 69.2 (1C, C-9), 66.9 (1C, C-11), 27.0, 25.5 (2 C13).

ESI-HRMS: *m/z* = 439.05139 $[M+H]^+$ (calculated *m/z* = 439.05139).

1-*O*-(4-(4-Iodophenyldiazenyl)benzene)-*rac*-glycerol (**12**):

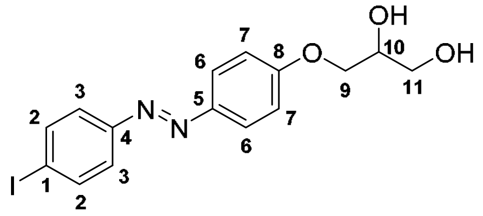

The isopropylidene-protected glycerol derivate **11** (643 mg, 1.47 mmol, 1.00 equiv) was dissolved in THF (20 mL). Aqueous hydrochloric acid (365 mg, 20.0 mL, 10.0 mmol, 0.5 M, 6.82 equiv) was added to the solution and the reaction mixture was stirred at room temperatur overnight. The reaction mixture was neutralized to pH = 7 with aq. NaOH solution (1 M). The reaction mixture was extracted with ethyl acetate (3x50 mL) and the solvent was removed under reduced pressure. The crude product was purified by column chromatography (cyclohexane/ ethyl acetate 1:1 → ethyl acetate/ methanol 1:1). 1-*O*-(4-(4-Iodophenyldiazenyl)benzene)-*rac*-glycerol (**12**) (498 mg, 1.25 mmol, 85% yield) was isolated as an orange solid.

$\boldsymbol{R_f}$ = 0.35 (cyclohexane/ ethyl acetate 1:1).

IR (ATR): $\tilde{\nu}$ =3294, 2929, 2973, 1600, 1562, 1240, 1033, 836, 549.

^{1}H NMR (600 MHz, DMSO-d$_6$) δ 7.97 – 7.92 (m, 2H, H-2), 7.92 – 7.87 (m, 2H, H-6), 7.65 – 7.60 (m, 2H, H-3), 7.17 – 7.11 (m, 2H, H-7), 5.03 (d, $^3J_{\text{OH-10–H-10}}$ = 5.1 Hz, 1H, OH-10), 4.72 (t, $^3J_{\text{OH-11–H-11}}$ = 5.7 Hz, 1H, OH-11), 4.13 (dd, $^2J_{\text{H-9a–H-9b}}$ = 9.9 Hz, $^3J_{\text{H-9a–H-10}}$ = 4.0 Hz, 1H, H-9a), 3.99 (dd, $^2J_{\text{H-9b–H-9a}}$ = 10.0 Hz, $^3J_{\text{H-9b–H-10}}$ = 6.2 Hz, 1H, H-9b), 3.87 – 3.80 (m, 1H, H-10), 3.47 (dd, $^3J_{\text{H-11–OH-11}}$ = 5.7 Hz, $^3J_{\text{H11-H10}}$ = 5.7 Hz, 2H, H-11).

^{13}C NMR (151 MHz, DMSO-d$_6$) δ 161.9 (1C, C-8), 151.3 (1C, C-4), 146.0 (1C, C-5), 138.3 (2C, C-2), 124.8 (2C, C-6), 124.1 (2C, C-3), 115.2 (2C, C-7), 97.9 (1C, C-1), 70.1 (1C, C-9), 69.8 (1C, C-10), 62.6 (1C, C-11).

ESI-HRMS: *m/z* = 399.02008 [M+H]$^+$ (calculated *m/z* = 399.02001).

1-*O*-(4-(4-Iodophenyldiazenyl)benzene)-2,3-di-*O*-hexadecanoyl-*rac*-glycerol (**14**):

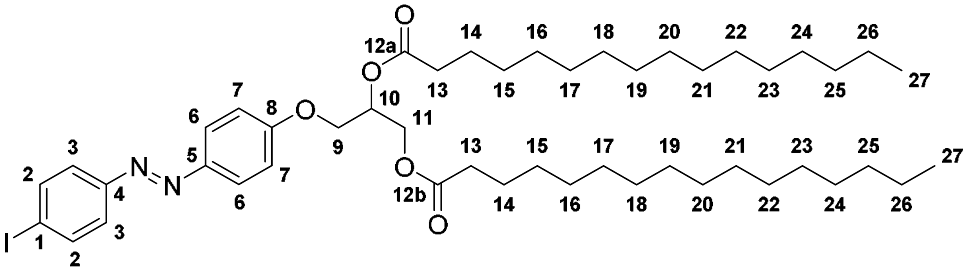

The azobenzene glycerol derivate **12** (270 mg, 678 µmol, 1.00 equiv) was dissolved in chloroform (30 mL). Hexadecanoic acid (**13**) (700 mg, 2.73 mmol, 4.03 equiv) and DMAP (85.1 mg, 697 µmol, 1.03 equiv) were added to the solution, followed by the addition of DCC (593 mg, 2.87 mmol, 4.24 equiv). The reaction mixture was heated to slight reflux (70 °C) for 48 h. The solvent was removed under reduced pressure and the crude product purified by column chromatography (cyclohexane → cyclohexane/ ethyl acetate 4:1 → ethyl acetate). 1-*O*-(4-(4-Iodophenyldiazenyl)benzene)-2,3-di-*O*-hexadecanoyl-*rac*-glycerol (**14**) (521 mg, 595 µmol, 88% yield) was isolated as a yellow powder.

$\boldsymbol{R_f}$ = 0.67 (cyclohexane/ ethyl acetate 4:1).

IR (ATR): $\tilde{\nu}$ = 2955, 2917, 2849, 1735, 1598, 1469, 1171, 840, 720, 546.

^{1}H NMR (600 MHz, CDCl$_3$) δ 7.93 – 7.88 (m, 2H, H-6), 7.86 – 7.82 (m, 2H, H-2), 7.63 – 7.59 (m, 2H, H-3), 7.03 – 6.99 (m, 2H, H-7), 5.45 – 5.40 (m, 1H, H-10), 4.48 – 4.43 (m, 1H, H-11a), 4.34 – 4.29 (m, 1H, H-11b), 4.22 – 4.18 (m, 2H, H-9), 2.39 – 2.29 (m, 4H, H-13), 1.68 – 1.58 (m, 4H, H-14), 1.38 – 1.14 (m, 48H, H-15-H-26), 0.91 – 0.85 (m, 6H, H-27).

^{13}C NMR (151 MHz, CDCl$_3$) δ 173.5 (1C, C-12b), 173.2 (1C, C-12a), 161.1 (1C, C-8), 152.2 (1C, C-3), 147.4 (1C, C-6), 138.4 (2C, C-2), 125.1 (2C, C-6), 124.4 (2C, C-3), 115.0 (2C, C-7), 97.0 (1C, C-1), 69.5 (1C, C-10), 66.5 (1C, C-9), 62.3 (1C, C-11), 34.4 (1C, C-13), 34.3 (1C, C-13), 32.1 (2C, C$_{aliphatic}$), 30.0 – 29.2 (m, 20C, C$_{aliphatic}$), 25.1 (2C, C-14), 22.9 (2C, C$_{aliphatic}$), 14.3 (2C, C-27).

C-15–C-26 are referred to as C$_{aliphatic}$ and could not be individually assigned.

ESI-HRMS: *m/z* = 875.47955 [M+H]$^+$ (calculated *m/z* = 875.47934).

1-*O*-(4-(4-(*S*-β-D-Glucopyranosyl)thiophenyldiazenyl)benzene)-2,3-di-*O*-hexadecanoyl-*rac*-glycerol (**1**):

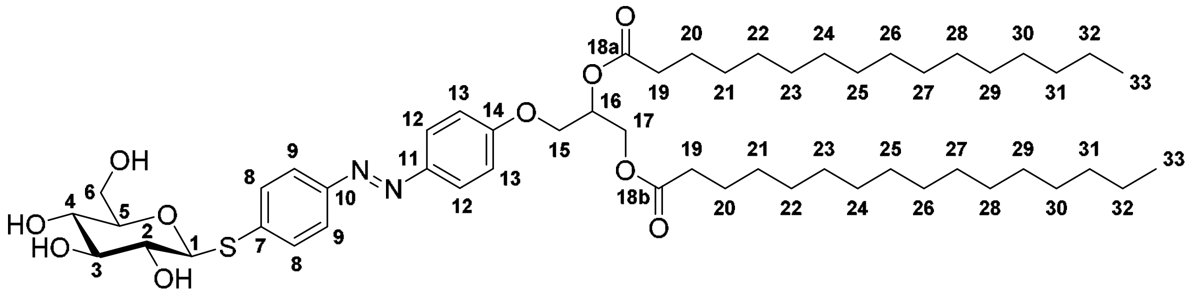

In an adaption to the protocol published by (Bruneau *et al.*, 2015), 1-thio-β-D- glucopyranose (**10**) (136 mg, 695 µmol, 3.80 equiv) was dissolved in a flame-dried round bottom flask by addition of dry 1,4-dioxane (15 mL) under N$_2$-atomsphere. After degassing the solution with three freeze-thaw cycles, the iodine substituted azobenzene derivative **14** (160 mg, 183 µmol, 1.00 equiv) and XantPhos Pd G3 (3.50 mg, 3.69 µmol, 0.02 equiv) were added to the flask. Triethylamine (18.9 mg, 26.0 µL, 187 µmol, 1.02 equiv) was added to the reaction mixture and the solution was stirred at room temperature overnight. Within the first minutes of the reaction, a discoloration of the reaction from yellow to reddish-brown was observed, in accordance to the observation described by Messaudi and coworkers, (Bruneau *et al.*, 2015) indicating the start of the reaction. As no complete conversion was observed, the reaction time was elongated overnight. After 18 h the reaction was extracted with brine (3x30 mL) and the solvent was removed under reduced pressure. The crude product was purified by column chromatography (cyclohexane/ ethyl acetate 1:1 → ethyl acetate). 1-*O*-(4-(4-(*S*-β-D-Glucopyranosyl)thiophenyldiazenyl)benzene)-2,3-di-*O*-hexadecanoyl-*rac*-glycerol (**1**) (116 mg, 123 µmol, 67% yield) was obtained as an orange solid.

R$_f$ = 0.45 (ethyl acetate).

IR (ATR): $\tilde{\nu}$ = 3366, 2955, 2917, 2849, 1735, 1727, 1602, 1241, 1043, 841, 550.

^{1}H NMR (600 MHz, $CDCl_3$) δ 7.89 – 7.84 (m, 2H, H-12), 7.79 – 7.75 (m, 2H, H-9), 7.59 – 7.54 (m, 2H, H-8), 7.00 – 6.95 (m, 2H, H-13), 5.45 – 5.37 (m, 1H, H-16), 4.74 (d, $^{3}J_{H\text{-}1\text{–}H\text{-}2}$ = 9.5 Hz, 1H, H-1), 4.45 (dd, $^{2}J_{H\text{-}17a\text{–}H\text{-}17b}$ = 12.0 Hz, $^{3}J_{H\text{-}17a\text{–}H\text{-}16}$ = 3.9 Hz, 1H, H-17a), 4.30 (dd, $^{2}J_{H\text{-}17b\text{–}H\text{-}17a}$ = 12.0, $^{3}J_{H\text{-}17b\text{–}H\text{-}16}$ = 6.1 Hz, 1H, H-17b), 4.20 – 4.13 (m, 2H, H-15), 3.97 – 3.85 (m, 2H, H-6), 3.74 – 3.63 (m, 2H, H-3, H-4), 3.54 – 3.48 (m, 1H, H-2), 3.48 – 3.42 (m, 1H, H-5), 2.37 – 2.30 (m, 4H, H-19), 1.66 – 1.58 (m, 4H, H-20), 1.36 – 1.19 (m, 48H, H-21–H-32), 0.90 – 0.84 (m, 6H, H-33).

^{13}C NMR (151 MHz, $CDCl_3$) δ 173.5 (1C, C-18b), 173.2 (1C, C-18a), 161.0 (1C, C-14), 152.0 (1C, C-10), 147.4 (1C, C-11), 135.6 (1C, C-7), 131.9 (2C, C-8), 125.1 (2C, C-12), 123.4 (1C, C-9), 115.0 (1C, C-13), 88.0 (1C, C-1), 79.6 (1C, C-5), 78.0 (1C, C-3), 72.4 (1C, C-2), 69.7 (1C, C-4), 69.5 (1C, C-16), 66.5 (1C, C-15), 62.4 (1C, C-17), 62.2 (1C, C-6), 34.4 (1C, C-19), 34.2 (1C, C-19), 32.1 (2C, $C_{aliphatic}$), 30.2 – 28.8 (20C, $C_{aliphatic}$), 25.1 (2C, C20), 22.8 (2C, $C_{aliphatic}$), 14.3 (2C, C-33).

C-21–C-32 are referred to as $C_{aliphatic}$ and could not be individually assigned.

ESI-HRMS: *m/z* = 943.60757 $[M+H]^+$ (calculated *m/z* = 943.60759).

NMR spectra of synthesized compounds

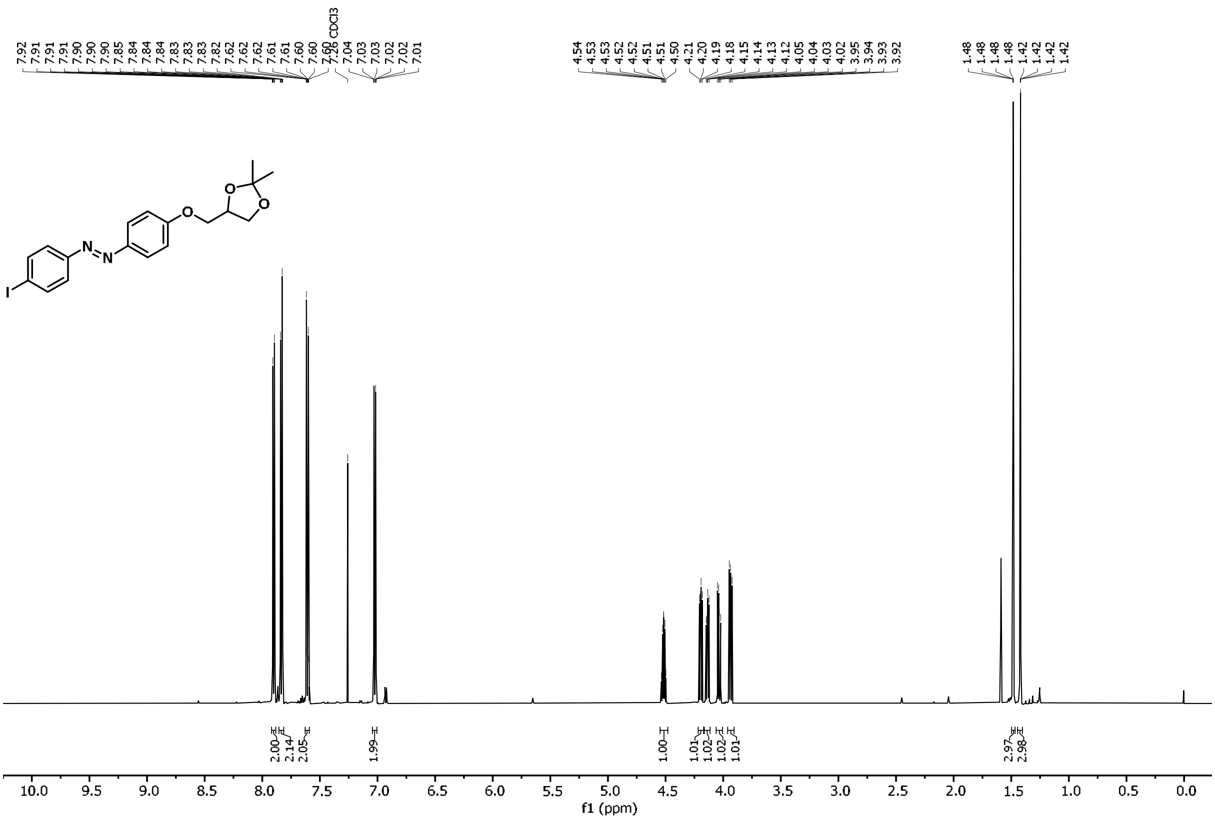

Figure S2 ^{1}H NMR spectrum of 1-*O*-(4-(4-iodophenyldiazenyl)benzene)-2,3-di-*O*-isopropylidene-*rac*-glycerol (**11**) (600 MHz, $CDCl_3$, 298 K).

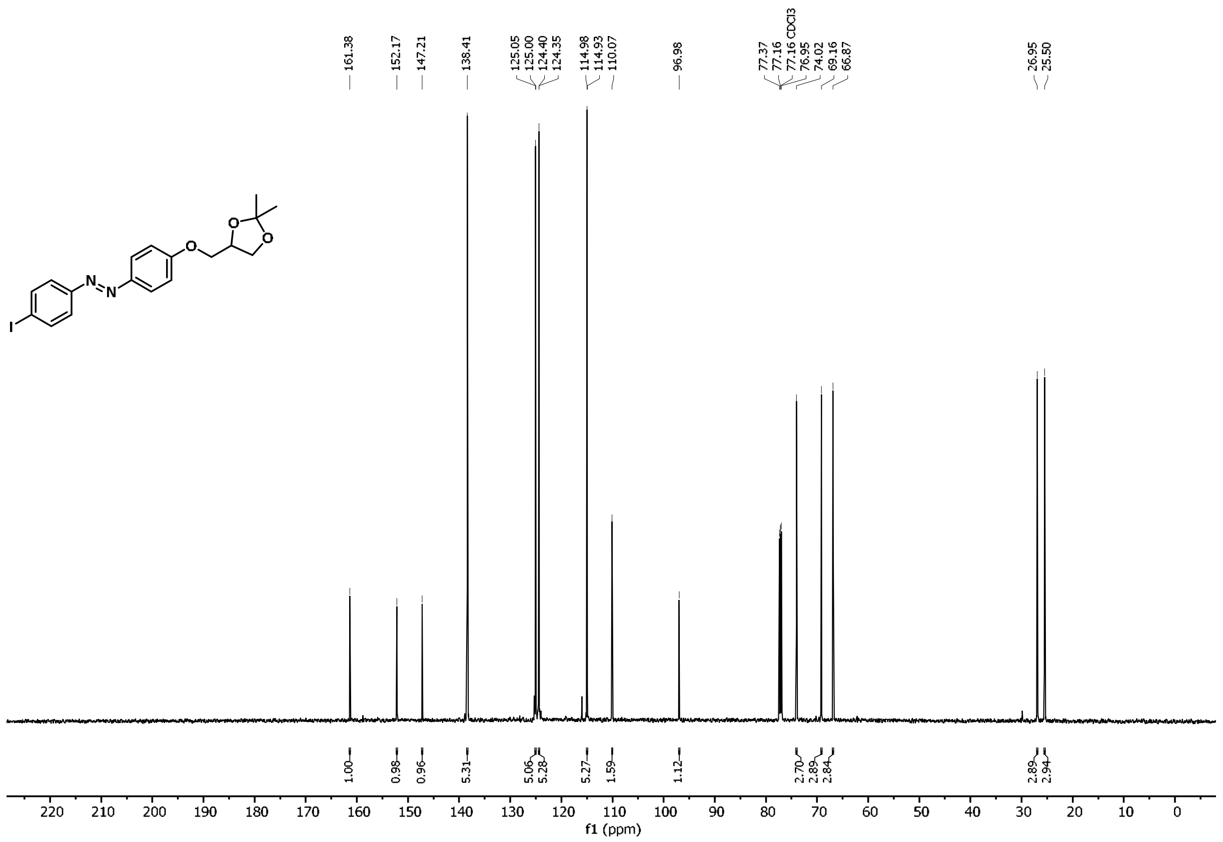

Figure S3 ^{13}C NMR spectrum of 1-O-(4-(4-iodophenyldiazenyl)benzene)-2,3-di-O-isopropylidene-rac-glycerol (**11**) (151 MHz, $CDCl_3$, 298 K).

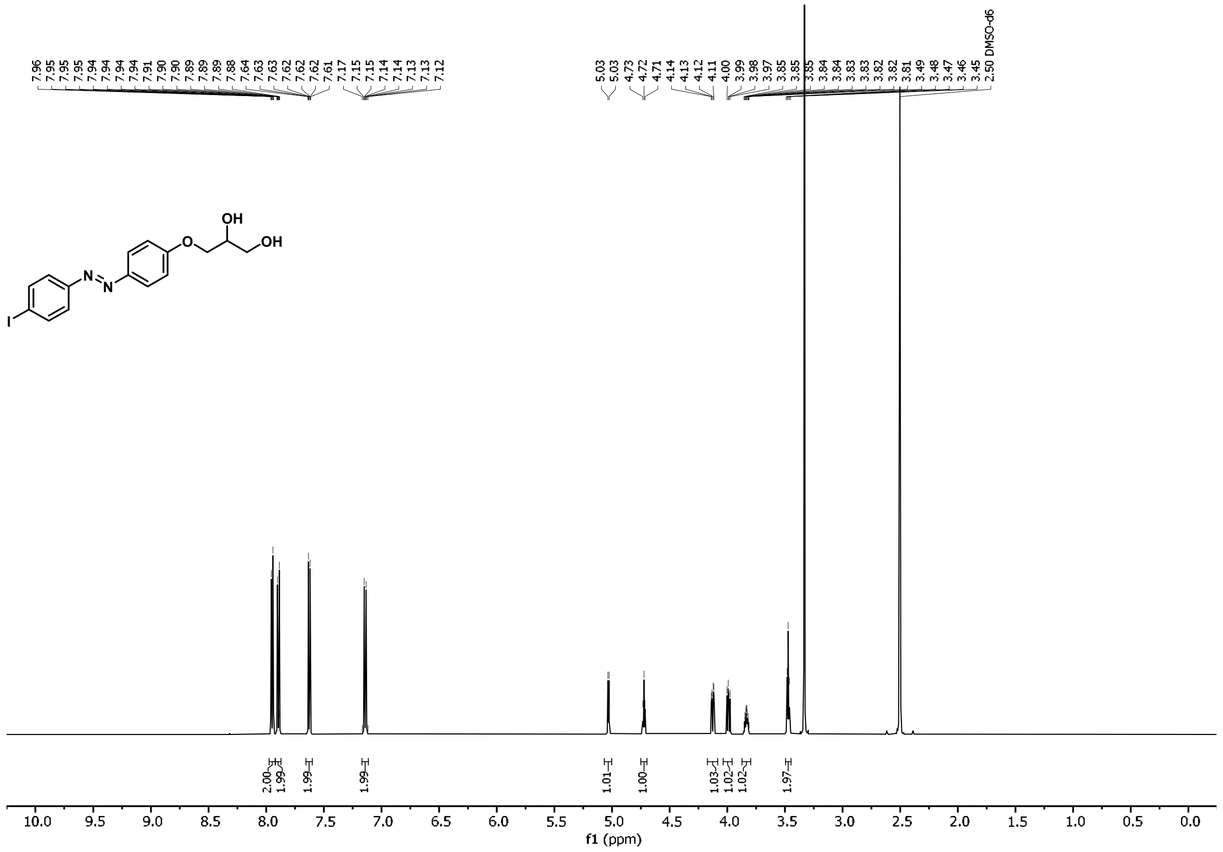

Figure S4 ^{1}H NMR spectrum of 1-*O*-(4-(4-iodophenyldiazenyl)benzene)-*rac*-glycerol (**12**) (600 MHz, DMSO-d$_6$, 298 K).

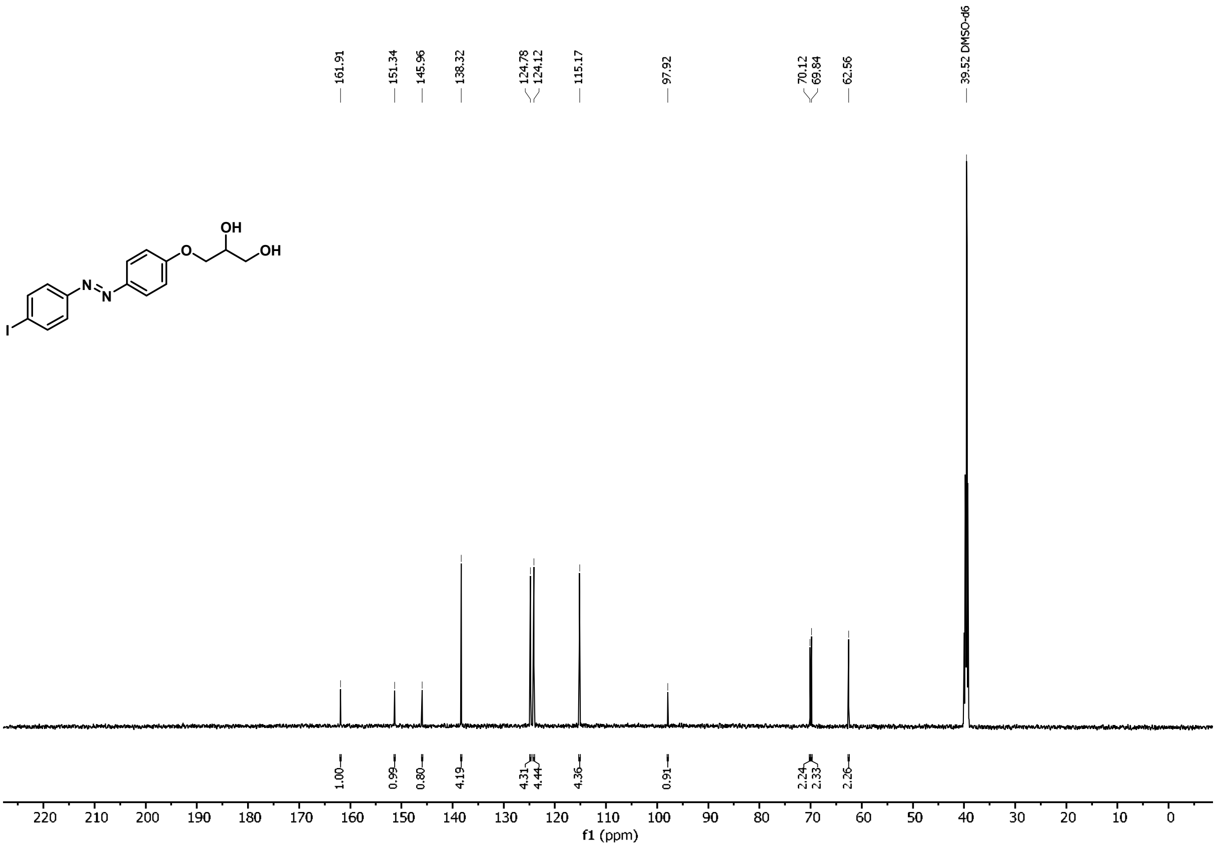

Figure S5 ^{13}C NMR spectrum of 1-*O*-(4-(4-iodophenyldiazenyl)benzene)-*rac*-glycerol (**12**) (151 MHz, DMSO-d$_6$, 298 K).

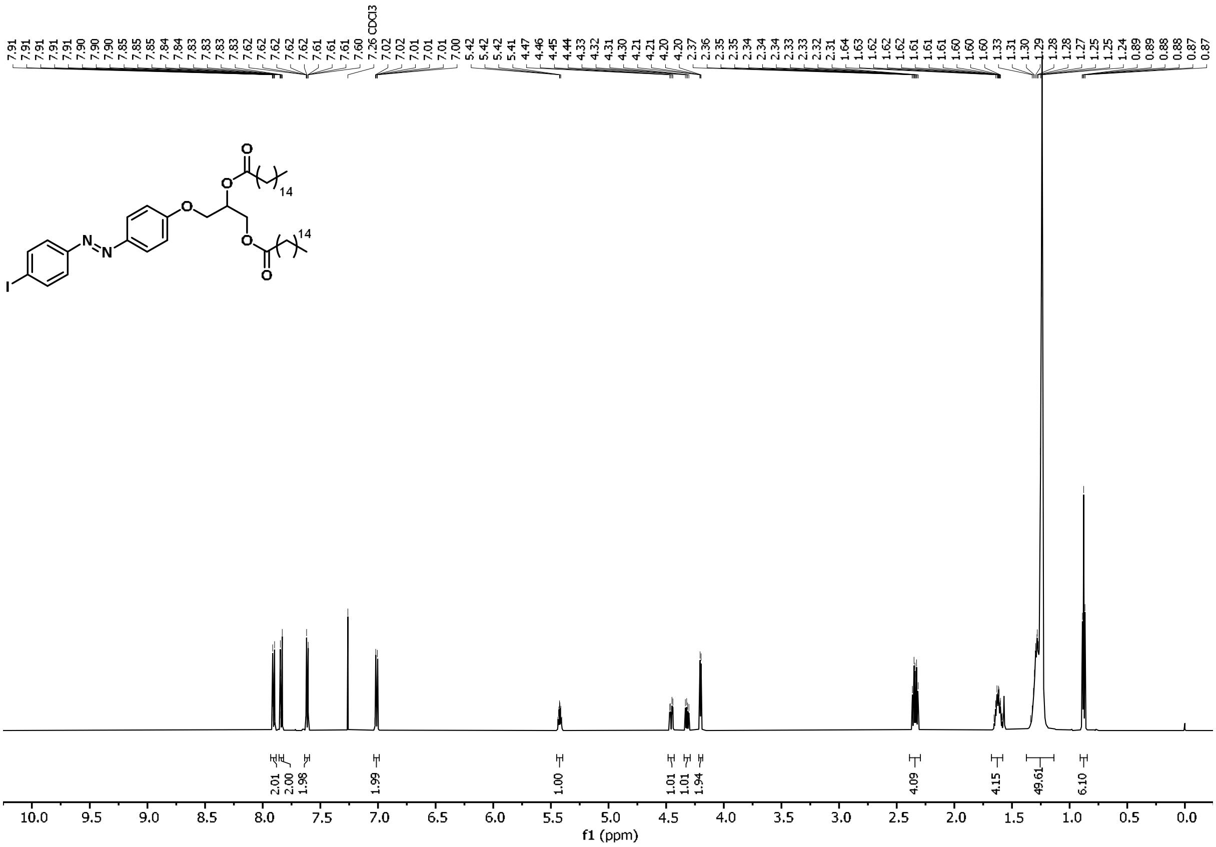

Figure S6 ^{1}H NMR spectrum of 1-*O*-(4-(4-iodophenyldiazenyl)benzene)-2,3-di-*O*-hexadecanoyl-*rac*-glycerol (**14**) (600 MHz, $CDCl_3$, 298 K).

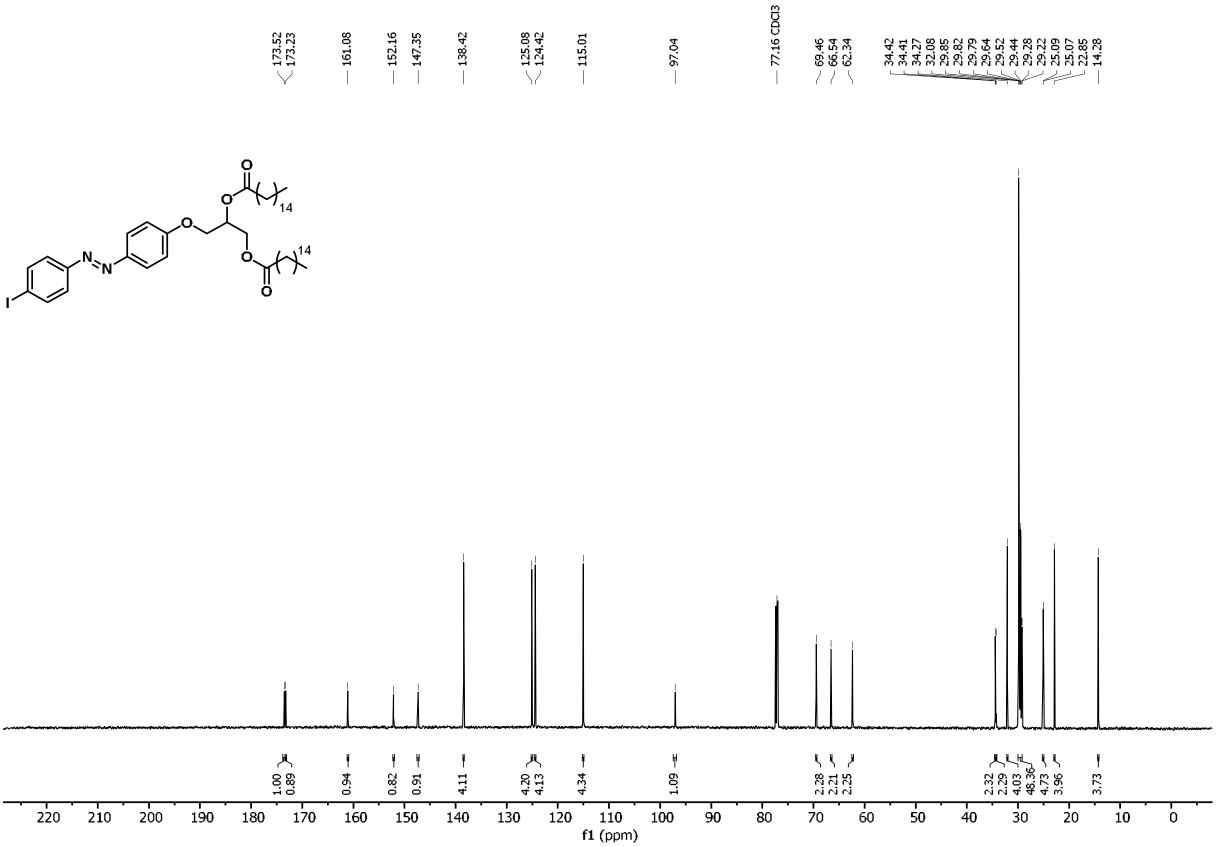

Figure S7 ^{13}C NMR spectrum of 1-*O*-(4-(4-iodophenyldiazenyl)benzene)-2,3-di-*O*-hexadecanoyl-*rac*-glycerol (**14**) (151 MHz, $CDCl_3$, 298 K).

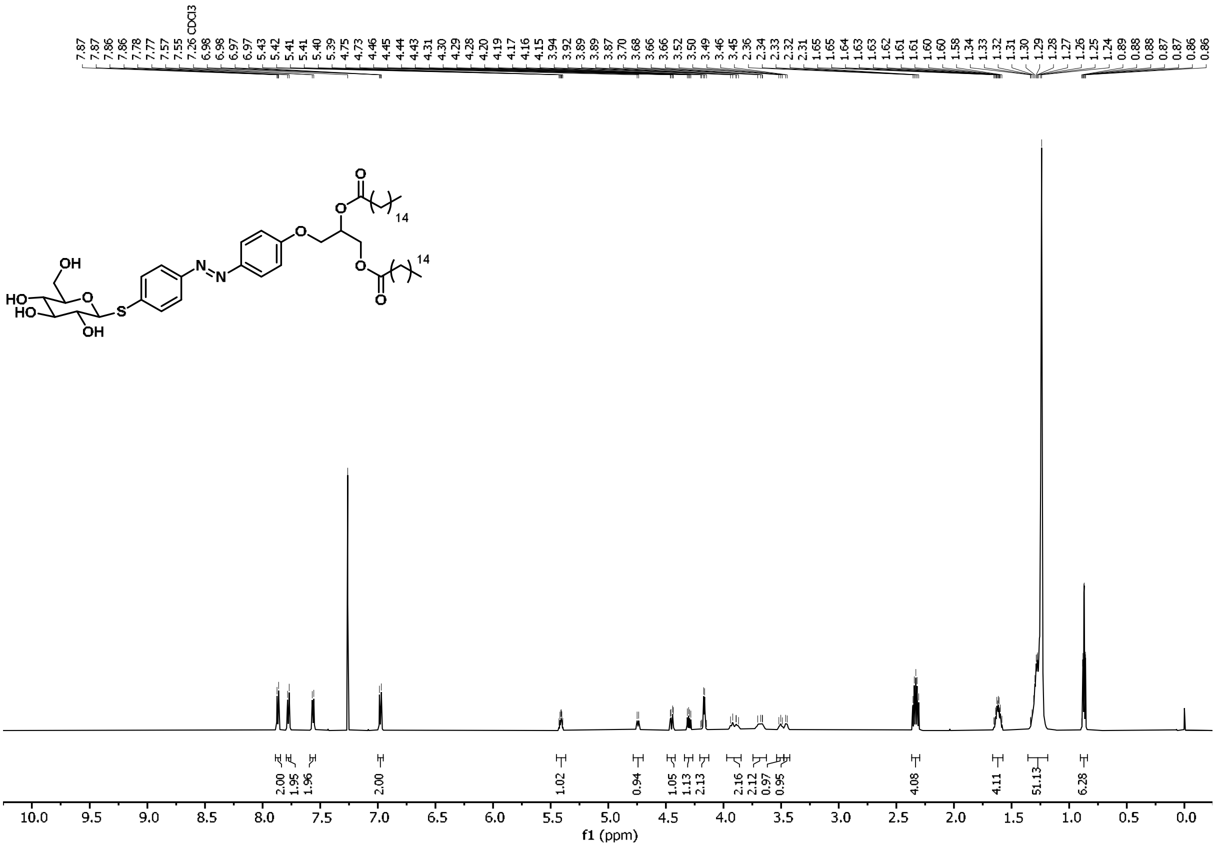

Figure S8 ^{1}H NMR spectrum of 1-*O*-(4-(4-(*S*-β-D-glucopyranosyl)thiophenyldiazenyl)benzene)-2,3-di-*O*-hexadecanoyl-*rac*-glycerol (**1**) (600 MHz, $CDCl_3$, 298 K).

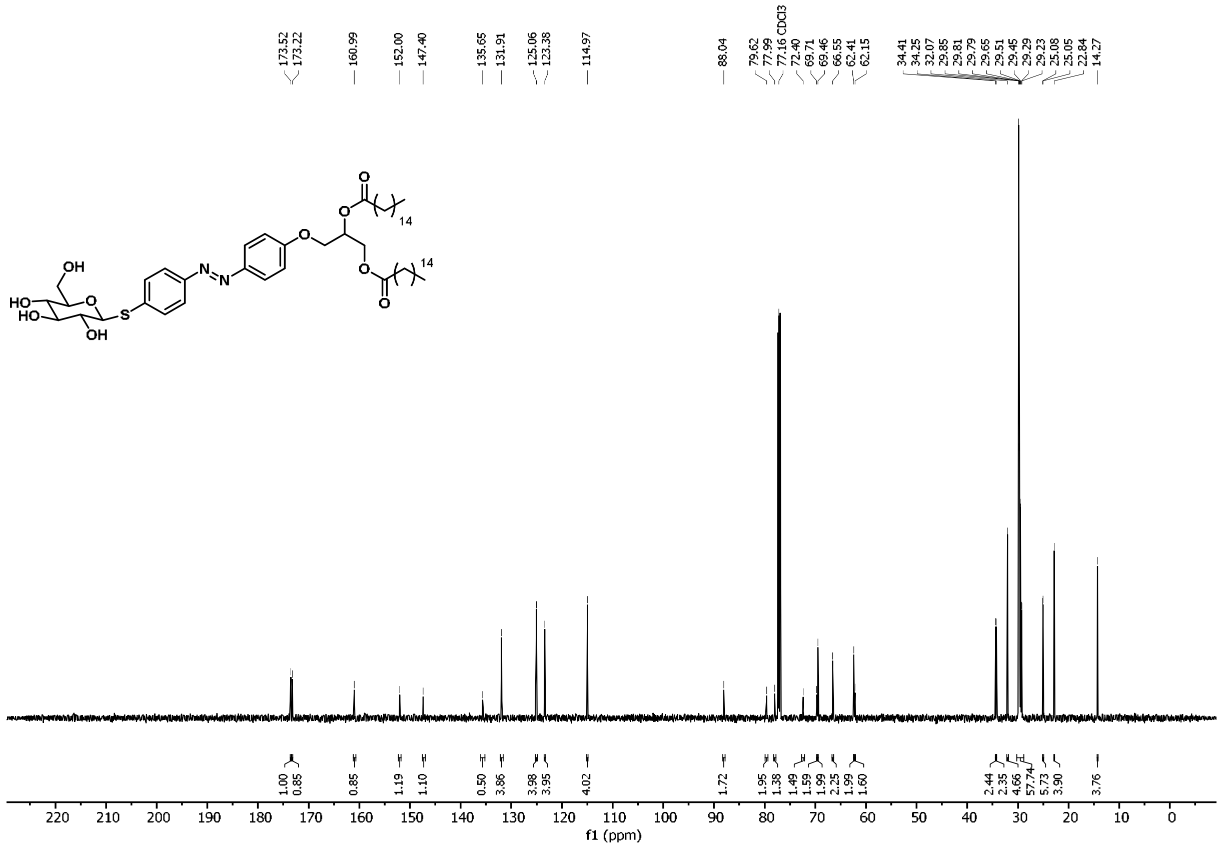

Figure S9 ^{13}C NMR spectrum of 1-*O*-(4-(4-(*S*-β-D-glucopyranosyl)thiophenyldiazenyl)benzene)-2,3-di-*O*-hexadecanoyl-*rac*-glycerol (**1**) (151 MHz, $CDCl_3$, 298 K).

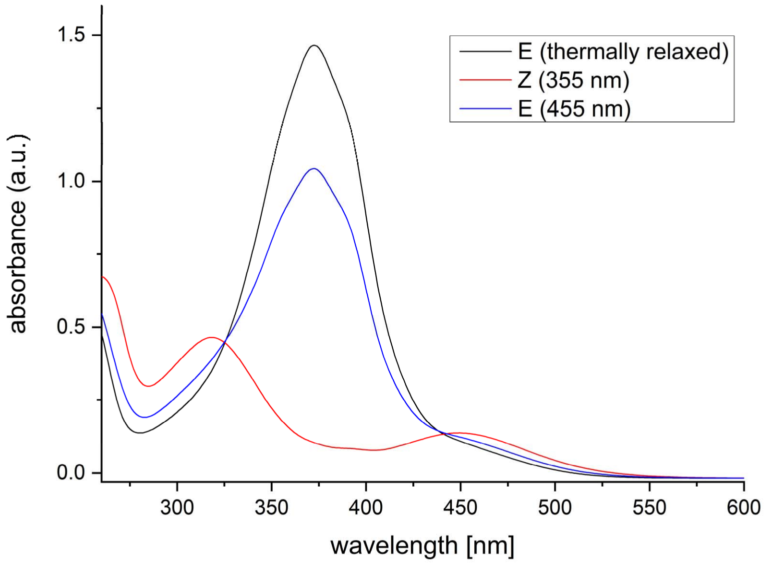

Figure S10 UV-VIS spectra of *trans*-**1** (blue) and *cis*-**1** (red) measured.

S2. Fitting different mesophases

As a first step to fit the SAXS data, a model describing possible structures, lamellar, hexagonal and bicontinuous cubic (*Pn3m*, *Im3m*, *Fm3m*, *Pm3n*, *Fd3m* and *Ia3d*), was plotted on top of the data to check visually for possible matches. The data was fitted between the wave vectors q = 0.6 and 3.5 nm^{-1}. The applied fit routine was previously used in (Hövelmann *et al.*, 2024). The model consists of multiple Gaussian-functions with variable amplitude $a_{1,2,\ldots,\mathrm{n}}$, peak width $\sigma_{1,2,\ldots,\mathrm{n}}$ and peak positions $p_{1,2,\ldots,\mathrm{n}}$ calculated based on the *d*-spacing parameter for the corresponding mesophases. The space groups are taken from (Hyde, 2001).

$$f(q) = a_1\, e^{-\frac{(q-p_1)^2}{2\sigma_1^2}} + \cdots + a_\mathrm{n}\, e^{-\frac{(q-p_\mathrm{n})^2}{2\sigma_\mathrm{n}^2}}$$

In most cases, only one matching structure was found. After identifying this best matching structure, the model was fitted again to the data allowing small deviations $\delta_{1,2,\ldots,\mathrm{n}}$ of the peak positions by maximum ± 0.06 nm^{-1} for each peak in the scattering data.

$$f(q) = a_1\, e^{-\frac{(q-(p_1+\delta_1))^2}{2\sigma_1^2}} + \cdots + a_\mathrm{n}\, e^{-\frac{(q-(p_\mathrm{n}+\delta_\mathrm{n}))^2}{2\sigma_\mathrm{n}^2}}$$

Based on the positions $p_\mathrm{n} + \delta_\mathrm{n}$ the *d*-spacing values were calculated and averages. Their deviation was used to determine the error of the *d*-spacing value.

Data such as for 90:10 or 80:20 DPPC:**2** show multiple peaks which cannot be described by a single mesophase and thus belong to different structures. In these cases, all possible combinations of two or three different mesophases were plotted on top of the data to check visually for matching peaks. The best matching combination was then fitted using the above described sequence. Thus, the overall fit consists of multiple Gaussian peaks belonging to the different mesophases as shown exemplarily in Fig. S11 for 20% of **2**.

For few data sets, both, the cubic *Pn3m* and *Im3m* structure, was found to fit the data reasonably well as for example shown in Fig. S12. In this case, the peak widths, deviations in the *d*-spacing values and the agreement with mesophases for both isomers and at other proportions were checked. In the case of 50:50 and 30:70 DPPC:**2** the *Im3m* structure was preferred as the peak widths, especially for the first peak, is smaller than for the *Pn3m* structure and the deviations in the peak positions from the theoretical positions are also smaller indicating a better match. In some cases, no unique solution of the structure could be identified as for example for the data on DPPC:**6** and DPPC:**7**. For these samples, the structure is labelled as not identified in Fig. 3 and no fit values are listed in Table S1. All other determined mesophases and their *d*-spacing are summarised in Table S1 for completeness.

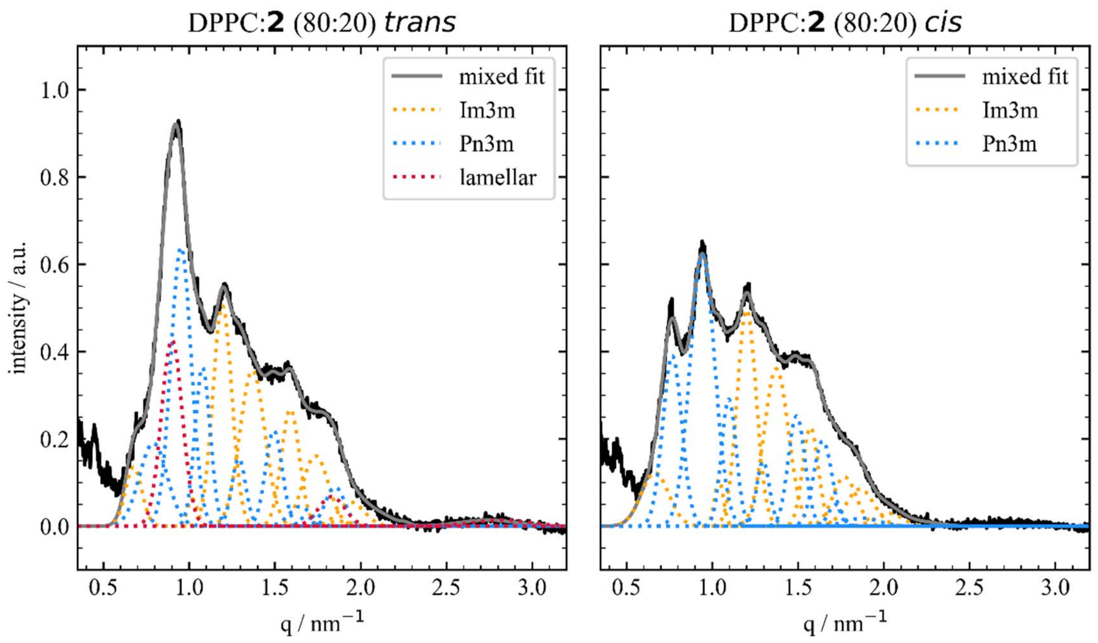

Figure S11 SAXS data obtained from 80% DPPC mixed with 20 % *trans*-**2** (left) and *cis*-**2** (right) shown with the fit (grey) of the sum of a lamellar (red), *Pn3m* (blue) and *Im3m* (orange) structure.

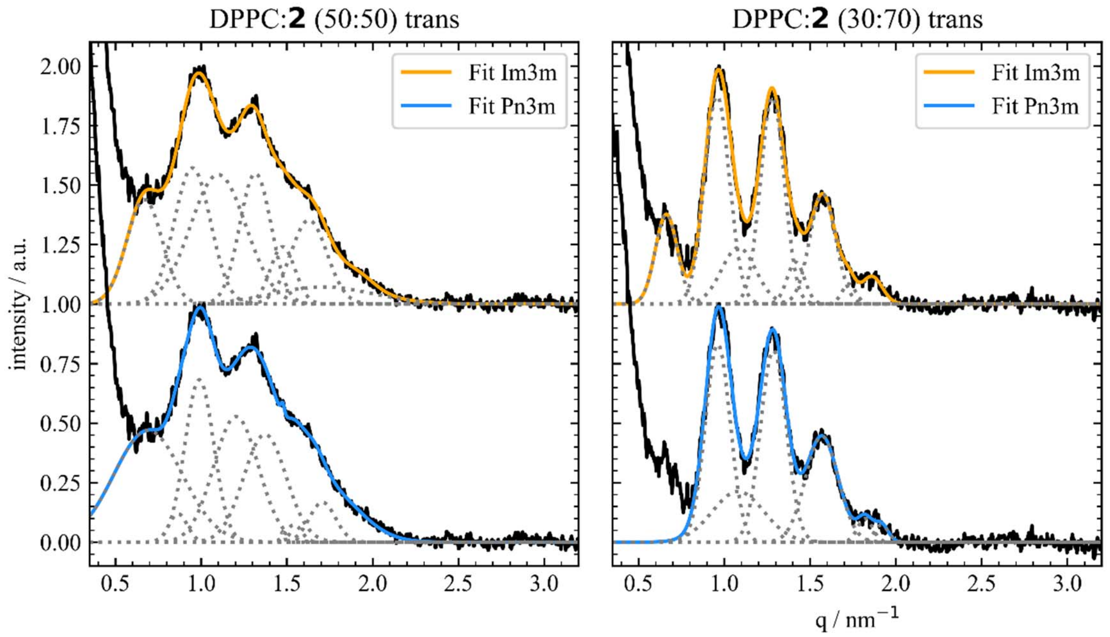

Figure S12 SAXS data obtained from DPPC mixed with *trans*-**2** in the proportion 50:50 (left) and 30:70 (right) together with the fits for the *Im3m* structure (top) and *Pn3m* structure (bottom).

Table S1 Mesophases and *d*-spacing parameters *d* derived from the SAXS data for pure DPPC and DLPC vesicles and mixed lipid aggregates together with the mass per volume with an estimated concentration error of 0.02 g/l.

T / °C	DPPC	density / g/l	mesophase	*d* / nm		
25	100	7.34	lamellar	6.34 ± 0.03		
			trans		*cis*	
T / °C	DPPC:**1**		mesophase	*d* / nm	mesophase	*d* / nm
21	97.5:2.5	3.70	lamellar	6.40 ± 0.02	lamellar	6.44 ± 0.01
21	95:5	3.72	lamellar	6.51 ± 0.01	lamellar	6.47 ± 0.02
21	90:10	3.78	lamellar	6.71 ± 0.04	lamellar	6.75 ± 0.04
21	50:50	4.19	1st order peak	6.67 ± 0.01	1st order peak	6.69 ± 0.01
21	0:100	4.72	1st order peak	6.78 ± 0.01	1st order peak	6.78 ± 0.01
			trans		*cis*	
T / °C	DPPC:**2**		mesophase	*d* / nm	mesophase	*d* / nm
25	95:5	7.41	lamellar	6.7 ± 0.09	Pn3m	10.4 ± 0.2
25	90:10	7.47	lamellar	6.8 ± 0.08	Pn3m	11.2 ± 0.4
21	80:20	3.80	lamellar	6.9 ± 0.09		
			Im3m	12.7 ± 0.3	Im3m	12.6 ± 0.4
			Pn3m	11.6 ± 0.3	Pn3m	11.7 ± 0.2
21	64:36	3.91	Im3m	12.4 ± 0.3	Im3m	12.5 ± 0.2
			Pn3m	11.3 ± 0.3	Pn3m	11.4 ± 0.3
25	50:50	8.00	Im3m	13.5 ± 0.1	Im3m	13.2 ± 0.1
25	30:70	8.27	Im3m	13.65 ± 0.5	Im3m	13.74 ± 0.5
25	0:100	8.67	lamellar	6.74 ± 0.05	lamellar	6.73 ± 0.04
			trans		*cis*	
T / °C	DPPC:**4**		mesophase	*d* / nm	mesophase	*d* / nm
25	90:10	7.64	lamellar	6.34 ± 0.05	lamellar	6.34 ± 0.05
25	80:20	7.93	lamellar	6.33 ± 0.04	lamellar	6.33 ± 0.04
			Pn3m	11.1 ± 0.2	Pn3m	11.2 ± 0.2

25	50:50	8.81	Pn3m	11.4 ± 0.1	Pn3m	11.3 ± 0.1
25	30:70	9.40	lamellar	8.0 ± 0.2	lamellar	8.0 ± 0.2
25	0:100	10.28	lamellar	8.0 ± 0.1	lamellar	8.0 ± 0.1
			trans		*cis*	
T / °C	DPPC:**5**		mesophase	*d* / nm	mesophase	*d* / nm
25	90:10	7.75	lamellar	6.42 ± 0.08	lamellar	6.57 ± 0.08
25	0:100	11.41	lamellar	8.63 ± 0.01	lamellar	8.62 ± 0.01
			lamellar	5.89 ± 0.02	lamellar	5.88 ± 0.02
T / °C	DLPC	density g/l	mesophase	*d* / nm		
25	100	3.11	lamellar	5.89 ± 0.01		
			trans		*cis*	
T / °C	DLPC:**2**		mesophase	*d* / nm	mesophase	*d* / nm
	95:5	3.17	lamellar	6.00 ± 0.01	lamellar	5.99 ± 0.01
	90:10	3.23	lamellar	6.02 ± 0.01	lamellar	6.00 ± 0.01
			Im3m	9.9 ± 0.2	Im3m	9.9 ± 0.2
			Pn3m	9.1 ± 0.2	Pn3m	9.2 ± 0.3
	80:20	3.35	Im3m	9.7 ± 0.2	Im3m	9.7 ± 0.2
			Pn3m	9.0 ± 0.1	Pn3m	9.0 ± 0.1
	50:50	3.72	Im3m	9.6 ± 0.2	Im3m	9.7 ± 0.2
			Pn3m	9.0 ± 0.1	Pn3m	9.1 ± 0.2
	30:70	3.97	Im3m	9.6 ± 0.2	Im3m	9.6 ± 0.2
	0:100	4.33	lamellar	6.74 ± 0.05	lamellar	6.73 ± 0.04
			trans		*cis*	
T / °C	DLPC:**3**		mesophase	*d* / nm	mesophase	*d* / nm
	95:5	3.20	lamellar	5.93 ± 0.01	lamellar	5.92 ± 0.01
	90:10	3.29	lamellar	5.93 ± 0.02	lamellar	5.93 ± 0.02
			Im3m	11.5 ± 0.8	Im3m	11.6 ± 0.8
			Pn3m	11.5 ± 0.7	Pn3m	11.7 ± 0.7
	80:20	3.47	lamellar	5.92 ± 0.02	lamellar	5.92 ± 0.02

		Im3m	11.6 ± 0.7	Im3m	11.5 ± 06
		Pn3m	11.1 ± 1.4	Pn3m	11.1 ± 1.4
50:50	4.00	Im3m	11.4 ± 1.2	Im3m	11.5 ± 1.6
		Pn3m	10.7 ± 1.2	Pn3m	10.8 ± 0.9
30:70	4.36	Im3m	11.2 ± 0.6	Im3m	11.0 ± 0.8
		Pn3m	11.2 ± 0.7	Pn3m	11.2 ± 0.7
0:100	4.89	p6m	7.6 ± 0.2	p6m	7.6 ± 0.2

S3. Fitting kinetics of photoswitching

Table S2 Mesophases and *d*-spacing parameters derived from the SAXS data for 97.5:2 and 95:5 DPPC:**1** at different times *t* after the start of illumination with 365 nm (*trans* to *cis*) and 455 nm (*cis* to *trans*).

		trans to cis		*cis to trans*	
t / s	DPPC:**1**	mesophase	*d* / nm	mesophase	*d* / nm
0	97.5:2.5	lamellar	6.442 ± 0.009	lamellar	6.407 ± 0.009
2	97.5:2.5	lamellar	6.445 ± 0.003		
5	97.5:2.5	lamellar	6.436 ± 0.009	lamellar	6.417 ± 0.009
10	97.5:2.5	lamellar	6.433 ± 0.008	lamellar	6.412 ± 0.010
20	97.5:2.5	lamellar	6.435 ± 0.007	lamellar	6.427 ± 0.006
30	97.5:2.5	lamellar	6.425 ± 0.008	lamellar	6.424 ± 0.010
40	97.5:2.5	lamellar	6.425 ± 0.012	lamellar	6.433 ± 0.009
50	97.5:2.5	lamellar	6.410 ± 0.005	lamellar	6.438 ± 0.005
60	97.5:2.5	lamellar	6.409 ± 0.009	lamellar	6.438 ± 0.007
120	97.5:2.5	lamellar	6.418 ± 0.004		
180	97.5:2.5	lamellar	6.407 ± 0.009		

		trans to cis		*cis to trans*	
t / s	DPPC:**1**	mesophase	*d* / nm	mesophase	*d* / nm
0	95:5	lamellar	6.512 ± 0.007	lamellar	6.475 ± 0.009
5	95:5	lamellar	6.512 ± 0.008	lamellar	6.485 ± 0.007

10	95:5	lamellar	6.504 ± 0.009	lamellar	4.495 ± 0.007
20	95:5	lamellar	6.498 ± 0.006	lamellar	4.489 ± 0.007
30	95:5	lamellar	6.491 ± 0.009	lamellar	4.497 ± 0.006
40	95:5	lamellar	6.482 ± 0.008		
45	95:5			lamellar	6.506 ± 0.006
50	95:5	lamellar	6.511 ± 0.007		
60	95:5	lamellar	6.479 ± 0.008	lamellar	6.501 ± 0.008
90	95:5			lamellar	6.518 ± 0.005
120	95:5	lamellar	6.486 ± 0.008		
180	95:5	lamellar	6.475 ± 0.009		

Table S3 Mesophases and *d*-spacing parameters derived from the SAXS data for 80:20 DPPC:**2** at different times *t* after the start of illumination with 365 nm (*trans* to *cis*) and 455 nm (*cis* to *trans*).

		trans to cis		*cis to trans*	
t / s	DPPC:**2**	mesophase	*d* / nm	mesophase	*d* / nm
0	80:20	lamellar	6.9 ± 0.2	Pn3m	11.6 ± 0.2
		Pn3m	11.6 ± 0.3	Im3m	12.6 ± 0.3
		Im3m	12.7 ± 0.3		
10	80:20	lamellar	6.9 ± 0.2		
		Pn3m	11.7 ± 0.3		
		Im3m	12.7 ± 0.3		
15	80:20			Pn3m	11.6 ± 0.3
				Im3m	12.6 ± 0.3
20	80:20	lamellar	6.8 ± 0.2		
		Pn3m	11.6 ± 0.3		
		Im3m	12.7 ± 0.3		
30	80:20	lamellar	6.9 ± 0.2	lamellar	6.9 ± 0.2
		Pn3m	11.6 ± 0.3	Pn3m	11.6 ± 0.3

		Im3m	12.6 ± 0.3	Im3m	12.6 ± 0.3
38	80:20	lamellar	6.9 ± 0.2		
		Pn3m	11.6 ± 0.3		
		Im3m	12.7 ± 0.3		
50	80:20	lamellar	6.9 ± 0.2		
		Pn3m	11.6 ± 0.3		
		Im3m	12.6 ± 0.3		
55	80:20	lamellar	6.8 ± 0.2		
		Pn3m	11.7 ± 0.3		
		Im3m	12.6 ± 0.3		
60	80:20	lamellar	6.8 ± 0.2	lamellar	6.9 ± 0.2
		Pn3m	11.8 ± 0.3	Pn3m	11.6 ± 0.3
		Im3m	12.7 ± 0.3	Im3m	12.8 ± 0.3
70	80:20	lamellar	6.9 ± 0.2		
		Pn3m	11.6 ± 0.3		
		Im3m	12.7 ± 0.3		
80	80:20	lamellar	6.9 ± 0.2		
		Pn3m	11.7 ± 0.3		
		Im3m	12.6 ± 0.3		
90	80:20	lamellar	6.8 ± 0.2	lamellar	6.8 ± 0.2
		Pn3m	11.6 ± 0.3	Pn3m	11.7 ± 0.3
		Im3m	12.6 ± 0.3	Im3m	12.7 ± 0.3
100	80:20	lamellar	6.8 ± 0.2		
		Pn3m	11.6 ± 0.3		
		Im3m	12.6 ± 0.3		
110	80:20	Pn3m	11.6 ± 0.3		
		Im3m	12.6 ± 0.2		
115	80:20	Pn3m	11.6 ± 0.2		
		Im3m	12.5 ± 0.3		
120	80:20	Pn3m	11.6 ± 0.2	lamellar	6.8 ± 0.2

		Im3m	12.6 ± 0.3	Pn3m	11.7 ± 0.3
				Im3m	12.7 ± 0.3
130	80:20	Pn3m	11.6 ± 0.3		
		Im3m	12.5 ± 0.3		
135	80:20			lamellar	6.9 ± 0.2
				Pn3m	11.7 ± 0.3
				Im3m	12.7 ± 0.3
150	80:20			lamellar	6.9 ± 0.2
				Pn3m	11.7 ± 0.3
				Im3m	12.7 ± 0.3
